# Development of a Person-Centred Coordinated Care Pathway in Swedish Healthcare for Low Back Pain

**DOI:** 10.5334/ijic.8940

**Published:** 2025-05-09

**Authors:** Allan Abbott, Malin Forsbrand, Thomas Torstensson, Ann-Charlotte Lindström, Gudrun Greim, Sammy Klaff, Åsa Niper, Marc Karlsson, Christian Simonsberg, Mimmi Engström, Tommy Olsson, Annelie Petersson, Per Ekman, Peter Försth, Gösta Ullmark, Steven J. Linton

**Affiliations:** 1 Unit of Physiotherapy, Department of Health, Medicine and Caring Sciences, Linköping University, Sweden; 2 Department of Orthopaedics in Linköping and Department of Biomedical and Clinical Sciences, Linköping University, Linköping, Sweden; 3 Department of Health, Blekinge Institute of Technology, Karlskrona, Sweden; 4 Department for quality and development, Region Blekinge, Karlskrona, Sweden; 5 Department of Public Health and Caring Sciences, Family Medicine and Preventive Medicine Section, Uppsala University, Uppsala, Sweden; 6 Department of Rehabilitation, NärRehab Primärvården Södra Älvsborg, Region Västra Götaland, Alingsås, Sweden; 7 Närhälsan Online, Region Västra Götaland, Sweden; 8 Trädgårdstorgets vårdcentral, Region Östergötland, Linköping, Sweden; 9 Division of Family Medicine and Primary Care, Department of Neurobiology, Care Sciences and Society, Karolinska Institutet, Stockholm, Sweden; 10 Vårdcentralen Hökarängen, Region Stockholm, Stockholm, Sweden; 11 Vårdcentralen Oxie, Region Skåne, Oxie, Sweden; 12 Smärtcentrum, Östra Hospital –Sahlgrenska University Hospital, Gothenburg, Sweden; 13 Nacka Rehabcentrum, Region Stockholm, Nacka, Sweden; 14 Patient representative, Swedish Rheumatism Association, Stockholm, Sweden; 15 Ryggkirurgiskt centrum, Region Stockholm, Stockholm, Sweden; 16 Department of Surgical Sciences, Orthopaedics and Handsurgery, Uppsala University, Uppsala, Sweden; 17 Department of Orthopedics, Gävle Hospital, Gävle, Sweden; 18 School of Law, Psychology and Social Work, Center for Health and Medical Psychology, Örebro University, Örebro, Sweden

**Keywords:** person-centred, care pathway, low back pain

## Abstract

**Introduction:**

This project aimed to develop a Person-Centred Co-ordinated Care (P3C) pathway for low back pain (LBP).

**Description:**

A national working group was formed consisting of representatives from all regional healthcare organisations in Sweden and included all relevant healthcare professions, academia, and patient organisations. A mixed method iterative design and consensus approach was applied in the development of the P3C pathway.

**Discussion:**

As a foundation, patient interviews along with a review of literature were conducted investigating the evidence base for healthcare interventions, earlier regional care programs/pathways and guidelines in Sweden as well as patient experiences and challenges with healthcare for LBP. Updated evidence-based clinical recommendations, tools supporting the practical use of the national P3C pathway and national healthcare data registry-based quality outcome indicators were then developed. Thereafter, an open consultation period provided review and feedback for final revisions and consensus.

**Conclusions:**

Essential factors for integrating best praxis according to scientific evidence and patient and healthcare professional perspectives were identified to establish a Swedish national P3C pathway for LBP. This provides a novel and innovative example of feasible methodology applicable in the international context. Future research will evaluate potential improvements in healthcare quality outcomes and effectiveness of dissemination and implementation strategies.

## Introduction

Low back pain (LBP) is a common condition that is often described as pain, discomfort, or stiffness in the lower back area, extending from the lower ribs to the lower gluteal folds, and/or related symptoms in one or both legs [1]. According to research, LBP has a lifetime prevalence of 51–84% and is the leading cause of years lost to disability [2]. It is also one of the most common reasons why people seek healthcare, and it has a significant socio-economic burden, including both direct healthcare costs and indirect societal costs [3,4]. Traditional biomedical models of care for LBP are poorly coordinated and outdated, which may contribute to these costs and impact on patients [5]. To address these challenges, an international call for action has been proposed to improve the quality and coordination of person-centred healthcare for LBP [6].

Healthcare redesign involves changing the way healthcare services are delivered to enhance their quality. Healthcare redesign is based on different quality improvement theories such as the theory of constraints [7], lean thinking [8], and complexity theory [9], quality management (TQM)/continuous quality improvement (CQI) [10,11,12] and re-engineering [13]. Redesign requires mapping existing care processes, elucidating problems and thereafter modifying these to optimise processes to provide fast and efficient care to patients, eliminating low value or redundant intervention, or risk of error, and achieving better outcomes [7]. Redesign methodology can also be used in the development of Person-Centred and Co-ordinated Care (P3C) pathways defined as healthcare processes guided by and organised effectively around the needs and preferences of individuals [14,15].

In 2018, the Swedish government and Swedish Council of Local Authorities and Regions collectively established a national system for knowledge-based management in healthcare [16]. This initiative is supported by 26 national program areas, with all 6 geographic region healthcare organisational clusters in Sweden represented by experts in respective domains. Additionally, national working groups were created within these program areas to action the national development of P3C pathways. This paper aims to describe the development of a national P3C pathway for LBP covering primary care processes until the patient can self-manage or transition to already established chronic care or secondary care pathways.

## Description of the care practice

### Methods

**Figure 1:**
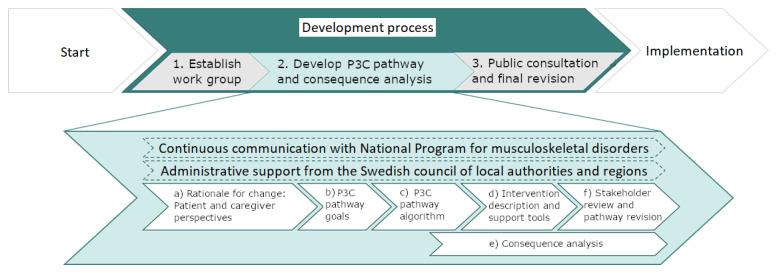
Development process of the P3C pathway for patients with LBP.

A mixed methods stepwise co-design approach guided by a healthcare redesign framework [7] was applied in the development of the P3C pathway for patients with LBP (Figure 1). The development of the P3C included the following steps:

1. Establish working group
  A national working group was formed through an independent nomination process from all 6 geographic region healthcare organisational clusters (Northern, Central, Stockholm/Gotland, Western, South-eastern, and Southern) encompassing 21 regional authorities in Sweden. The selected working group contained clinical experts in LBP (>15–40 years’ experience) from all 6 of the geographic region healthcare organisational clusters in Sweden and consisted of 4 physiotherapists, 3 general practitioners, 2 spinal surgeons, 1 rehabilitation medicine physician (pain and psychiatric specialist), 1 psychologist, 1 chiropractor, 1 occupational therapist as well as 2 patient partner representatives. This composition of healthcare professions proportionally reflected the direct access workforce involved in primary care of patients with LBP in Sweden. Academic credentials in the national working group also included 6 members with PhDs (3 physiotherapists, 2 spinal surgeons, 1 psychologist) and 2 with professorships (1 physiotherapist, 1 psychologist). The working group had regular communication with a steering committee coordinating a national program area for musculoskeletal disorders and received administrative support in the form of process management and information technology specialist consultations provided by the Swedish Council of Local Authorities and Regions. The national working group are the authors of the current paper.
2. Develop P3C pathway and consequence analysis
  - *Rationale for change: Patient and care giver perspectives:*
    To provide a basis for describing existing healthcare services for patients with LBP, the working group reviewed literature describing regional care programs, pathways and guidelines in Sweden and internationally. This provided a basis for discussion in a 1-day workshop format to determine perspectives within the working group regarding the current barriers and facilitators care givers face in providing healthcare for patients with LBP. Survey literature investigating the impact of LBP from a patient perspective (n = 9642) in Sweden and internationally [17] as well as a systematic review of qualitative literature on patient experiences and challenges with healthcare for LBP were also reviewed by the working group [18]. In addition to the perspectives from the 2 patient partner representatives (1 man, 1 women) in the national working group, in-depth interviews were performed by the working group. These were of 60-minute duration and conducted on patients with LBP (n = 3) volunteering as representatives from patient organisations and had experience of healthcare for LBP. These included a 50-year-old women with recurring episodes of LBP, 60-year-old women with chronic LBP and a 81-year-old women with chronic LBP and multimorbidity. Interviews were audio recorded and transcribed through naturalised verbatim by 2 working group members. The following semi-structured interview guide was utilised in the interviews:
    - Can you describe how LBP started for you?
    - What has your contact with the healthcare system been like regarding LBP?
      - What did it look like for you (e.g. contact with the healthcare telephone service “1177”, primary care, referral and so on) and how did you experience the different steps in the care pathway?
    - What are the biggest challenges you as a patient with LBP have faced in contact with the healthcare system?
    - What do you think are the biggest challenges for the healthcare system regarding treatment of patients with LBP?
    - How could healthcare services for patients with LBP be improved based on your experiences as a patient?
      - What goals would you like a healthcare service pathway for LBP to achieve?
      - A rehabilitation plan, also known as a patient contract, is a central part of a healthcare pathway. What should it highlight?
  An inductive approach to reflexive qualitative thematic analysis was used by the working group to analyse the literature and patient interviews [19]. This through familiarisation with the data by reading and rereading the literature and patient interview transcripts, collaboratively generating initial codes for meaningful phrases and thereafter creating initial themes and diagramming relationships between themes. This process went through 3 iterations of review within the working group to finalise the themes and relationships between themes identifying the experiences and challenges of healthcare for LBP as a rationale for developing the P3C pathway. The in-depth patient interviews were considered to reach saturation due to recurring themes from other mixed method data reviewed [17,18].

b) *P3C pathway goals:*
  After identifying the themes underlining the rationale for the P3C pathway, a 1-day workshop format was performed by the working group to align these themes to specific goals for the P3C pathway for LBP. Healthcare quality process and outcome indicators were constructed to allow monitoring of the goals of the P3C pathway for LBP in the healthcare regions nationally according to methods described by Stelfox & Straus [20,21].
c&d) *P3C pathway algorithm, pathway description table and support tools:*
  To provide clarity regarding the current evidence base for assessment and treatment interventions for LBP, the working group analysed previously published literature. The search strategy involved combining search terms describing the condition and interventions and entering them into the Cochrane library, Swedish Council on Health Technology and Assessment (SBU), and Pubmed databases. The search strategy was limited to systematic review literature and extended to individual randomised controlled trials if no systematic reviews existed. Additional selection criteria were applied to prioritise the most recent Cochrane or SBU reviews over other types of systematic reviews. Assessment of methodological quality of systematic reviews was conducted individually by at least 2 authors using the AMSTER checklist and a third author was consulted to solve discrepancies in AMSTER assessments [22]. Thereafter, the working group constructed best practice recommendations regarding assessment and treatment of LBP which provided the basis for developing the P3C pathway algorithm and accompanying pathway description table. Additional tools supporting the clinical interventions described by the P3C pathway were also developed such as red flag triage and referral acuity tools, recommendations for investigating yellow and blue flags (psychosocial and work-related factors), subjective and objective assessment proformas, recommendations for patient reported measures and ICD-10 diagnosis codes, a patient information brochure and rehabilitation plan/contract proforma. A total of 7x1-day workshops were performed by the working group in steps c-d.
e) *Consequence analysis*: Possible consequences of implementation of the P3C were explored by the working group during a 1-day workshop. Benefits versus risks for patients, ethical aspects, organisational aspects, costs, staff competency requirements, influence on other knowledge support systems and consequences regarding the follow-up of data indicators for the P3C pathway were explored.
f) *Stakeholder review and P3C revision*: Stakeholders including the 1 patient organisation Swedish (Rheumatism Association – Scope covering all musculoskeletal conditions causing pain and inflammation), 4 health profession organisations (National Physiotherapy Association, National Nurses Association, National Chiropractic Association; National Orthopaedic Association) and 4 national healthcare specialty groups (National program area for rehabilitation, habilitation and insurance medicine; National primary healthcare council; National working group for chronic pain; National working group for Sweden’s pharmaceutical committee), and 1 clinical and research (associate professor) expert in LBP were tasked with providing feedback on the P3C pathway June-July 2022. A total of 2x1-day workshops were performed by the working group to revise the P3C pathway in accordance with stakeholder feedback.

3. Open public consultation and final revision: The P3C pathway was open for public consultation during November 2022 – January 2023. Feedback was provided by a total of 12/21 healthcare regions (Kronoberg, Västra Götaland, Stockholm, Västmanland, Värmland, Västerbotten, Blekinge, Östergötland, Halland, Dalarna, Västernorrland, Örebro län), 7 national healthcare specialty groups (National program area for rehabilitation, habilitation and insurance medicine; National primary healthcare council, National program area for rheumatic diseases; National program area for acute healthcare; National working group for chronic pain; National working group for Sweden’s pharmaceutical committee), 4 regional healthcare specialty groups (Central Sweden’s & Stockholm’s regional program area for musculoskeletal disorders; Stockholm regional program area for rheumatic diseases; Uppsala regional primary care), 4 health professional organisations (Swedish association of physiotherapists; Swedish McKenzie Institute, Swedish rehabilitation medicine association; Swedish nursing association), 1 community healthcare district (Flen), 1 national government department (E-health), 1 university research centre (Gothenburg person-centred care GPCC), 1 company (Boston Scientific Nordic AB) and 2 individuals (Private practice physiotherapists). The P3C national workgroup held a 1-day workshop to discuss and revise the P3C pathway based on the public consultation feedback.

### Results

**Figure 2:**
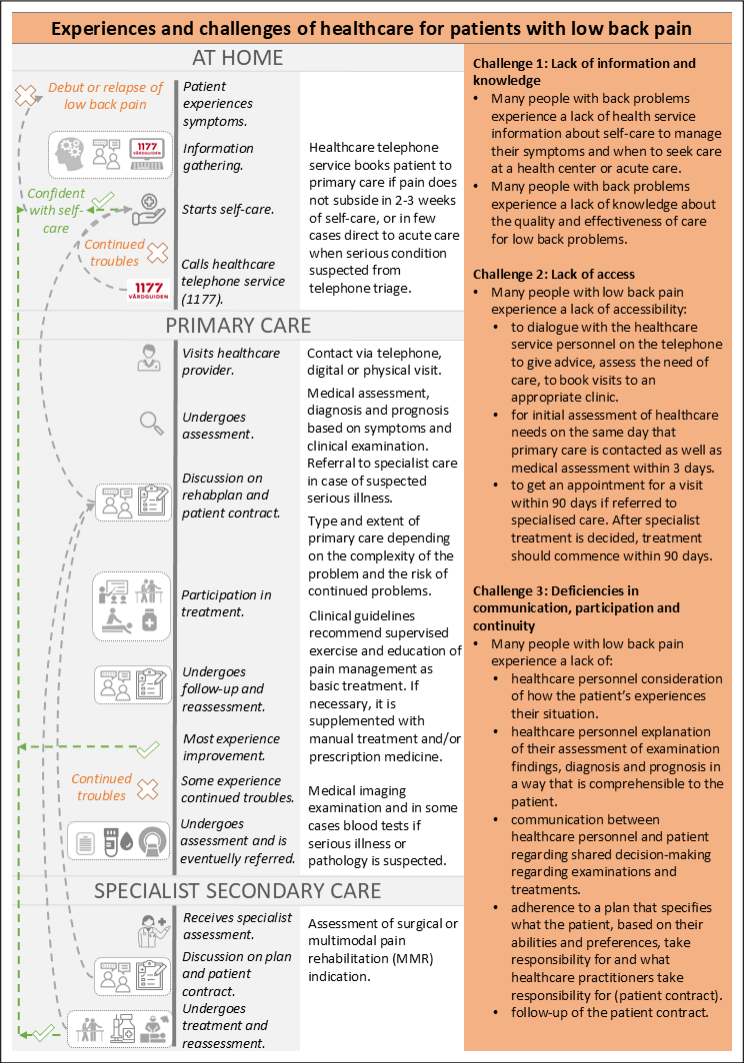
Rationale for change: Summary of patient and caregiver perspectives resulting from methods described in 2a) of the P3C pathway development process.

**Figure 3:**
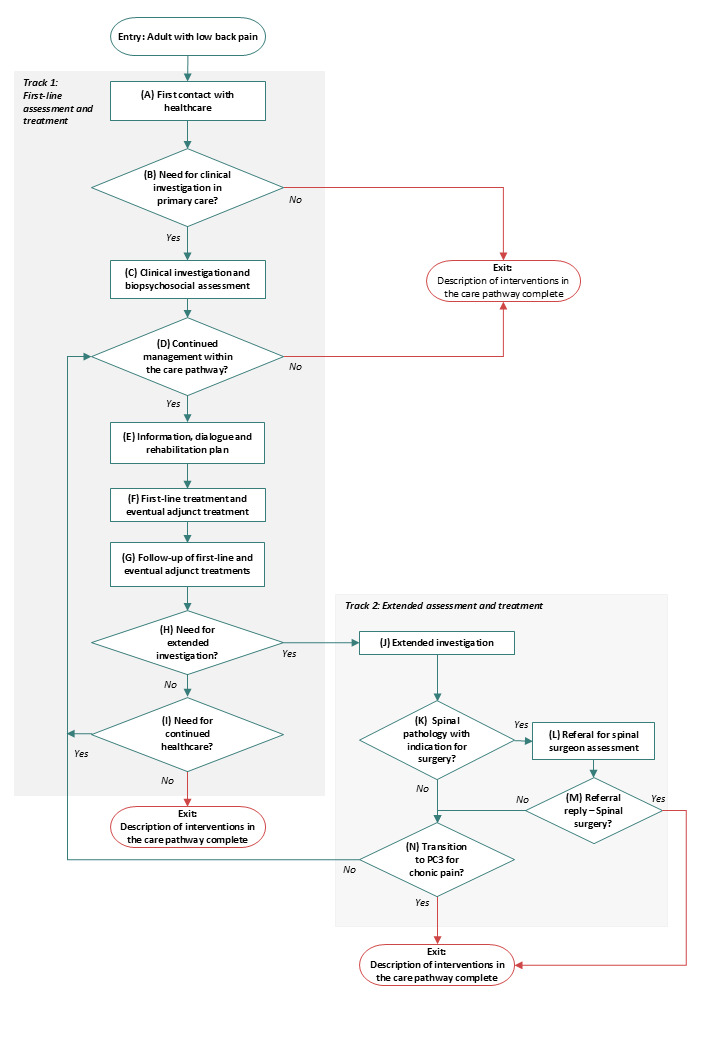
P3C pathway algorithm.

**Figure 4:**
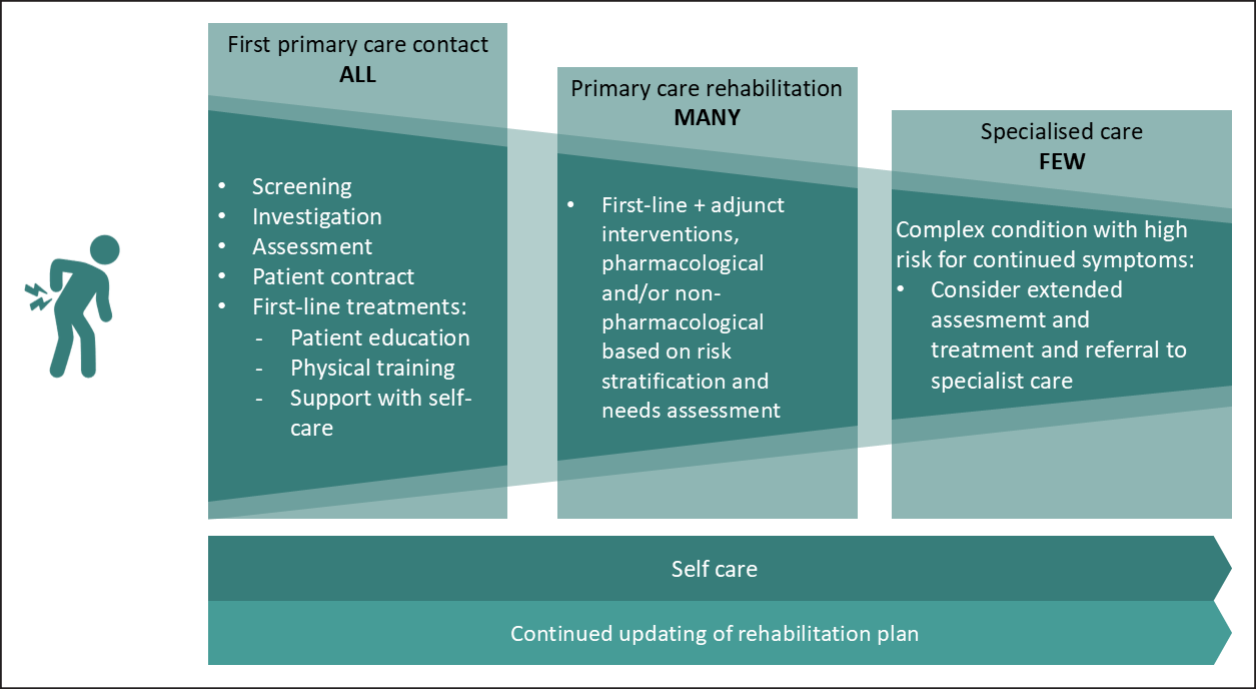
Brief summary of assessment and treatment interventions in the P3C pathway.

- *Rationale for change – Patient and care giver perspectives:*
  Experiences and challenges of healthcare for patients with LBP were identified as a basis for designing the P3C pathway. In Figure 2, column 1 describes identified positive and negative patient experiences, column 2 lists the activities and procedures that are common for the patient, column 3 describes existing healthcare processes and care giver perspectives and column 4 describes the challenges of healthcare for patients with LBP.
- *P3C pathway goals:*
  The goal of the care pathway is for the patient to experience/achieve:
  1. good continuity and coordination of the treatment episode.
  2. good participation in their care/treatment.
  3. good knowledge of one’s state of health.
  4. increased ability to function, be active and/or work.
  5. increased health-related quality of life through reduced discomfort and/or improved ability to manage any remaining ailments.
  To follow up the P3C pathway goal attainment, quality indicators were developed based on accessible data sources and are displayed in Appendix A.
- *P3C pathway algorithm*:
  The algorithm displayed in Figure 3 describes a stepwise approach to patient assessment and treatment. The algorithm suggests a basis for what should be performed and in what order, while allowing healthcare personnel to adjust specific content for patient individualisation and situational factors. The inclusion criteria for using the algorithm are adults contacting primary healthcare because of problems suspected to originate from the lumbar spine, regardless of duration of symptoms and whether it is a new onset or relapse. A person can be included in the care process on repeated occasions. Exclusion or exit from the P3C pathway occurs if the patient, after assessment, and where applicable after treatment, is considered: a) to be able to cope with self-care b) need urgent care c) need supplementary assessment and care in another pathway or specialised care d) refrains from the treatment measures offered, after information and dialogue about the expected effect or e) is assessed to not benefit from further course of care.
- *P3C pathway description table and support tools*:
  As an aid to clinicians in understanding the P3C algorithm, each step in the algorithm is explained in detail in Appendix B. Evidence-based recommendations on assessment and treatment of LBP for which the algorithm is based upon are summarised in Appendix C. Figure 4 briefly summarises the P3C pathway interventions. All patients who have problems that are suspected to be related to the lumbar spine should undergo a screening for red flags (Appendices D&E) as well as individual assessment and suggestions regarding interventions at the appropriate level of care. Interventions can vary from advice on self-care to recommendations for specialised care. Patients who have had problems for more than two to three weeks should be offered a clinical examination, screening of yellow and blue flags (Appendices F and G), assessment of patient-reported measures (Appendix H) and receive a diagnosis (Appendix I) at the first contact/s with primary care. If screening shows a low to moderate risk of long-term problems, the patient has a good prognosis, and the symptoms can regress with the help of self-care and first-line treatment. If the screening shows a moderate to high risk of long-term problems and where serious pathology is not suspected, the patient is expected to benefit from individualised multimodal rehabilitation interventions that can be provided in primary care. A smaller proportion of patients need specialised care. All patients can benefit from following advice on self-care (Appendix J) at all stages of the P3C pathway. Planned interventions are documented in a rehabilitation plan/patient contract (Appendix K) in which the interventions planned by the healthcare provider and the patient are described concretely and clearly. The patient is expected to be well informed and involved in proposed assessment and treatment steps in the algorithm.
  Implementation tools such as videos describing the algorithm pathway steps exemplified by patient cases are available online [23]. The P3C pathway in its entirety is also published on the national knowledge support website [24] for healthcare personnel along with national P3C pathways and knowledge support tools for other diagnoses. The goals of the P3C pathway are achievable by the healthcare system providing patients with LBP with the right assessment and care at the right time, through an individualised investigation and treatment.
- *Consequence analysis*:
  A consequence analysis resulted in the following list of potential risks and benefits of the P3C pathway and facilitators and barriers for its national implementation:
  1. National evidence-based clinical guidelines for the care of patients with LBP have been lacking in Sweden, even though internationally there are several published clinical guidelines. Guidelines from other countries recommend applying the basis of the biopsychosocial model to assess and treat LBP, which is not a fully established clinical approach in Sweden.
  2. To be able to work from a biopsychosocial approach, healthcare providers need to incorporate a wider patient perspective early in the subjective and objective examination. One way to structure the examination is to start from screening of “red, yellow and blue flags” to adapt the treatment based on the individual’s needs. The screening utilises patient’s medical history and the examination to provide a basis for decisions about the right treatment for the individual.
  3. Screening can identify which patients can cope with first-line treatment. The purpose of the first-line treatment is to stimulate strategies for self-care with the help of patient education and physical exercise to reduce the need for use of analgesic medications and treatment methods where the patient becomes a passive recipient of the treatment.
  4. If the patient is identified with a “red flag” and suspicion of serious pathology exists, the patient needs to undergo further investigation without long waiting times. The investigation may involve examination with magnetic resonance imaging (MRI) or assessment within specialised care. Today, there are large regional differences in access to MRI, which means that new MRI equipment or increased staffing on existing equipment may be needed to ensure patient safety.
  5. The P3C pathway highlights that patients identified with “yellow and blue flags” need an in-depth investigation and may need collaboration between several healthcare professionals and in multimodal rehabilitation. That possibility is not available currently in all healthcare regions.
  6. For the P3C pathway to be followed, new skills and organisational changes may be required some healthcare region areas. To ensure good implementation, training may be needed for various healthcare providers in preparation in the regions for receiving the P3C pathway.
  7. Previous studies have shown that adherence to evidence regarding primary care interventions for LBP varies between different primary care clinics within two regions in Sweden, which can be generalised to other regions in Sweden.
  8. If healthcare regions fail to implement the P3C pathway due to lack of resources or personnel, it is possible for clinicians to work according to the pathway with help of tools and videos presented in the P3C pathway.

## Discussion

The aim to develop a national P3C pathway for LBP has been achieved engaging key national stakeholders and provides a novel and innovative example of feasible methodology applicable in the international context. To build a person-centred foundation, it was of importance that patient partner representatives were involved in all steps in the working group as well as patient interviews along with review of existing literature investigating patient experiences and challenges with healthcare for LBP [25]. In addition, utilising existing context specific regional care programs, pathways, and guidelines along with recent systematic reviews and meta-analyses of international literature was of importance to provide a basis for updating evidence-based clinical recommendations as well as constructing process algorithms and tools supporting the practical use of the national P3C pathway. The use of national healthcare data registry-based indicators were of importance to provide a coordinated system to evaluate care pathway quality outcomes over time [26]. In a final step, an open consultation period provided a forum for critical review and feedback from a societal wide perspective including the general public, patient organisations, healthcare professional organisations, academia and policy makers as a final iteration of revision and consensus [27].

The P3C pathway for LBP is well aligned with international literature for clinical practice guidelines and care pathways [28,29,30,31,32]. Similarities include the focus on systematic assessment, risk stratification, imaging only when needed, patient education, cognitive behavioural approach, active physical therapies, simple analgesics first, judicious use of complex medicines, structured follow-up and timely referrals. Another similarity is the further application of risk stratification to direct more comprehensive intervention and support for those at higher risk of poor outcomes, while underscoring that the most common pathway is for first-line treatments and self-management. However, there are also some innovative differences in contrast with previous literature. Firstly, the concept of a national P3C pathway for LBP provides an innovative example of how to coordinate healthcare and empower patients via patient-centred communication and through building upon a traditional healthcare giver use of rehabilitation plans by the addition of a specific patient behavioural contracts to help attain P3C goals. Secondly, the P3C pathway recognises that many patients seeking care have recurrent pain and decreased work ability and therefore promotes a broad assessment of red, yellow and blue flags to stratify the patients to the right care at the right time rather than basing healthcare on limited conceptions of acute, subacute and chronic pain. Finally, a major strength with the P3C pathway is that a national macro-level agreement is in place with all regional healthcare organisations on a meso-level as well as healthcare providers on a micro-level committing to P3C pathway implementation and follow-up.

Initial discussions regarding developing and implementing P3C pathways in Sweden have previously met some skepticism, voicing concern for lack of evidence for such processes, risk of reducing professional freedom, individual assessments resulting in over-diagnosing, overtreatment and displacement effects [33,34]. Leaving expressed room for individual clinical considerations and adjustments is therefore an important part of the P3C pathway methodology. The P3C pathway provides a standardised policy scaled for national implementation but it’s fundamental person-centred and healthcare integration design allows inherent flexibility for context specific regional healthcare organisation and local primary care clinics adaptations. For example, some primary care clinics may have a multidisciplinary team ready to support patients at high risk for poor outcome while others may not and therefore need to coordinate with other facilities. Providing support for meso-level and micro-level organisational implementation is a key factor in achieving actual patient benefit. Ensuring actual improvement in patient care is however complex and using solely guideline-based approaches have been shown to have both potential limitations and harms besides desired benefits [35]. As LBP is an area where the strength of evidence informing best practice is still partially limited, there is a risk of recommending ineffective treatments. To mitigate this risk, the P3C pathways are subject to continuous revisions. National guidelines can also conflict with locally modified guidelines, creating confusion and frustration. In Sweden, national guidelines have been updated according to P3C pathways to avoid conflicting recommendations on a national level, and further updates of local guidelines will coincide with regional and local implementation processes.

The general challenge of implementing P3C pathways in primary healthcare is that results are dependent on the actions of a large number of individual clinical healthcare personnel interpreting the pathway similarly and adapting practice with their context. Future implementation into regional and local healthcare organisations will follow the Translating Research into Practice (TRIP) framework [36]. This involves each healthcare region in Sweden creating regional and local multidisciplinary working groups performing gap analyses, implementing local action plans and administrating future engage-educate-execute-evaluate cycle management of the P3C pathway in collaboration with the national working group. Future research will evaluate potential improvements in healthcare quality outcomes and processes as well as barriers and facilitators for dissemination and implementation strategies.

## Conclusion

Essential factors for integrating best praxis according to scientific evidence and patient and healthcare professional perspectives were identified to establish a Swedish national P3C pathway for LBP. This provides a novel and innovative example of feasible methodology applicable in the international context. Future research will evaluate potential improvements in healthcare quality outcomes and processes as well as barriers and facilitators for dissemination and implementation strategies.

## Additional File

The additional file for this article can be found as follows:

10.5334/ijic.8940.s1 Appendices. Appendix A–K.
