# Supplementary material for: Development of a Person-Centred Coordinated Care Pathway in Swedish Healthcare for Low Back Pain
Source: Int J Integr Care. 2025 May 9;25(2):8. doi: 10.5334/ijic.8940 (PMC12063581; doi:10.5334/ijic.8940)
Supplement: Appendices. — Appendix A–K. [file ijic-25-2-8940-s1.zip › ijic-8940_abbott-s1.docx]

Appendix A. P3C pathway goal follow-up data indicators

| **The goal of the care pathway is for the patient to experience/achieve:** | **Indicator type** | **Indicator formulation** | **Source** | **Numerator** | **Denominator** | **National data coverage and completeness** |
| --- | --- | --- | --- | --- | --- | --- |
| 1. Good continuity and coordination of the treatment episode. | Result outcome | Proportion of patients with LBP who have a National Patient Survey dimension score above 70% regarding continuity and coordination. | National patient survey* – Domain = Continuity and coordination (Questions 5,29,6,13,12,28,27). | Number of patients who have an NPE domain score above 70%. | Number of patients per year who have responded to NPE after completing the course of care and have an ICD-10 diagnosis code listed in Appendix G. | Development indicator based on national PREM with 100% coverage. Completeness dependent on patient response rate. |
|  | Process outcome | Annual average healthcare costs for patients with LBP. | Healthcare region databases. | Total healthcare costs per year according to the “cost per patient model” for patients described by the denominator. | Number of patients per year who have an ICD-10 diagnosis code listed in appendix G. | 100% coverage, 100% completeness. |
|  | Process outcome | Proportion of patients with LBP who receive assessment within 3 days of first contact. | Healthcare region databases. | Number of patients as described by the denominator who have received medical assessment by licensed personnel within 3 days after the patients first contact with primary care. | Number of patients per year who have an ICD-10 diagnosis code listed in appendix G. | 100% coverage, 100% completeness. |
|  | Process outcome | Proportion of patients with LBP who have undergone first-line treatment prior to diagnostic imaging. | Healthcare region databases. | Number of patients as described by the denominator who have received medical imaging. | Number of patients per year who have an ICD-10 diagnosis code listed in appendix G. | Indicator based on medical imaging codes in medical record system. 100% coverage. 100% completeness. |
|  | Process outcome | Proportion of patients with LBP who have undergone first-line treatment before referral to specialised care in orthopaedics | Healthcare region databases. | Number of patients as described by the denominator who have undergone first-line treatment before referral to specialised care in orthopaedics | Number of patients per year who have an ICD-10 diagnosis code listed in appendix G. | Development indicator based on use of treatment codes for first-line LBP interventions in medical record system. 100% coverage. Completeness dependent on clinician use of search term. |
| 2. Good participation in their care/treatment. | Result outcome | Proportion of patients with LBP who have a National Patient Survey dimension score above 70% regarding participation. | National patient survey* – Domain = Participation and involvement. (Questions 17,16,23,15). | Number of patients who have an NPE domain score above 70%. | Number of patients per year who have responded to NPE after completing the course of care and have an ICD-10 diagnosis code listed in appendix G. | Development indicator based on national PREM with 100% coverage. Completeness dependent on patient response rate. |
| 3. Good knowledge of one's state of health. | Result outcome | Proportion of patients per year who feel they have good knowledge of their health status after a period of treatment for low back problems. | National Patient Survey* (NPE) – Domain = information and knowledge (Questions 4,19,21,22). | Number of patients who have an NPE domain score above 70%. | Number of patients per year who have responded to NPE after completing the course of care and have an ICD-10 diagnosis code listed appendix G. | Development indicator based on national PREM with 100% coverage. Completeness dependent on patient response rate. |
| 4. Increased ability to function, be active and/or work. | Process outcome | Average number of sickness benefit days per year for patients with LBP who have received treatment. | The Swedish Social insurance agency. | Number sickness benefit days per year for patients as described by the denominator. | Number of patients per year who have an ICD-10 diagnosis code listed in appendix G. | 100% coverage, 100% completeness. |
|  | Process outcome | Proportion of patients with LBP who receive risk assessment based on Start Back Screening Tool or ÖMPSQ-short version. | Healthcare region databases. | Number of patients as described by the denominator that have received documented risk assessment for persistent LBP based on Start Back Screening Tool or ÖMPSQ-short version. | Number of patients per year who have an ICD-10 diagnosis code listed in appendix G. | Development indicator based on “risk assessment” search term in medical record system. 100% coverage. Completeness dependent on clinician use of search term. |
| 5. Increased health-related quality of life through reduced discomfort and/or improved ability to manage any remaining ailments. | Result outcome | Proportion of patients with LBP who have had reduced pain estimated according to NRS/VAS after basic treatment. | Healthcare region databases. | Number of patients as described by the denominator with a reduction in pain of at least 2 on the 10-point NRS/VAS scale from NEW VISIT to after the DATE of the first registration of the codes included in first-line care. | Number of patients per year who have received first-line treatment for an ICD-10 diagnosis code listed in appendix G. | Development indicator based on “NRS/VAS pain” search term in medical record system. 100% coverage. Completeness dependent on clinician use of search term. |

*Nationella patientenkäten, Sverige Landsting och Regioner i Samverkan (2015). Rapport Analysuppdrag: Modellutveckling, utvärdering samt tidigare studier och enkäter. https://skr.se/download/18.40c889381840e60521aa1a14/1668006119029/Rapport%20Analysuppdrag_Modellutveckling,%20utv%C3%A4rdering%20samt%20tidigare%20studier%20och%20enk%C3%A4ter_2015.pdf
