# Supplementary material for: Development of a Person-Centred Coordinated Care Pathway in Swedish Healthcare for Low Back Pain
Source: Int J Integr Care. 2025 May 9;25(2):8. doi: 10.5334/ijic.8940 (PMC12063581; doi:10.5334/ijic.8940)
Supplement: Appendices. — Appendix A–K. [file ijic-25-2-8940-s1.zip › ijic-8940_abbott-s1.pdf]

Appendix A. P3C pathway goal follow-up data indicators

The goal of the care pathway is for the patient to experience/achieve:	Indicator type	Indicator formulation	Source	Numerator	Denominator	National data coverage and completeness
1. Good continuity and coordination of the treatment episode.	Result outcome	Proportion of patients with LBP who have a National Patient Survey dimension score above 70% regarding continuity and coordination.	National patient survey* – Domain = Continuity and coordination (Questions 5,29,6,13,12,28,27).	Number of patients who have an NPE domain score above 70%.	Number of patients per year who have responded to NPE after completing the course of care and have an ICD-10 diagnosis code listed in Appendix G.	Development indicator based on national PREM with 100% coverage. Completeness dependent on patient response rate.
	Process outcome	Annual average healthcare costs for patients with LBP.	Healthcare region databases.	Total healthcare costs per year according to the “cost per patient model” for patients described by the denominator.	Number of patients per year who have an ICD-10 diagnosis code listed in appendix G.	100% coverage, 100% completeness.
	Process outcome	Proportion of patients with LBP who receive assessment within 3 days of first contact.	Healthcare region databases.	Number of patients as described by the denominator who have received medical assessment by licensed personnel within 3 days after the patients first contact with primary care.	Number of patients per year who have an ICD-10 diagnosis code listed in appendix G.	100% coverage, 100% completeness.
	Process outcome	Proportion of patients with LBP who have undergone first-line treatment prior to diagnostic imaging.	Healthcare region databases.	Number of patients as described by the denominator who have received medical imaging.	Number of patients per year who have an ICD-10 diagnosis code listed in appendix G.	Indicator based on medical imaging codes in medical record system. 100% coverage. 100% completeness.
	Process outcome	Proportion of patients with LBP who have undergone first-line treatment before referral to specialised care in orthopaedics	Healthcare region databases.	Number of patients as described by the denominator who have undergone first-line treatment before referral to specialised care in orthopaedics	Number of patients per year who have an ICD-10 diagnosis code listed in appendix G.	Development indicator based on use of treatment codes for first-line LBP interventions in medical record system. 100% coverage. Completeness dependent on clinician use of search term.
2. Good participation in their care/treatment.	Result outcome	Proportion of patients with LBP who have a National Patient Survey dimension score above 70% regarding participation.	National patient survey* – Domain = Participation and involvement. (Questions 17,16,23,15).	Number of patients who have an NPE domain score above 70%.	Number of patients per year who have responded to NPE after completing the course of care and have an ICD-10 diagnosis code listed in appendix G.	Development indicator based on national PREM with 100% coverage. Completeness dependent on patient response rate.
3. Good knowledge of one's state of health.	Result outcome	Proportion of patients per year who feel they have good knowledge of their health status after a period of treatment for low back	National Patient Survey* (NPE) – Domain = information and knowledge (Questions	Number of patients who have an NPE domain score above 70%.	Number of patients per year who have responded to NPE after completing the course of care and have an ICD-10 diagnosis code listed appendix	Development indicator based on national PREM with 100% coverage. Completeness dependent on patient response rate.

		problems.	4,19,21,22).		G.	
4. Increased ability to function, be active and/or work.	Process outcome	Average number of sickness benefit days per year for patients with LBP who have received treatment.	The Swedish Social insurance agency.	Number sickness benefit days per year for patients as described by the denominator.	Number of patients per year who have an ICD-10 diagnosis code listed in appendix G.	100% coverage, 100% completeness.
	Process outcome	Proportion of patients with LBP who receive risk assessment based on Start Back Screening Tool or ÖMPSQ-short version.	Healthcare region databases.	Number of patients as described by the denominator that have received documented risk assessment for persistent LBP based on Start Back Screening Tool or ÖMPSQ-short version.	Number of patients per year who have an ICD-10 diagnosis code listed in appendix G.	Development indicator based on “risk assessment” search term in medical record system. 100% coverage. Completeness dependent on clinician use of search term.
5. Increased health-related quality of life through reduced discomfort and/or improved ability to manage any remaining ailments.	Result outcome	Proportion of patients with LBP who have had reduced pain estimated according to NRS/VAS after basic treatment.	Healthcare region databases.	Number of patients as described by the denominator with a reduction in pain of at least 2 on the 10-point NRS/VAS scale from NEW VISIT to after the DATE of the first registration of the codes included in first-line care.	Number of patients per year who have received first-line treatment for an ICD-10 diagnosis code listed in appendix G.	Development indicator based on “NRS/VAS pain” search term in medical record system. 100% coverage. Completeness dependent on clinician use of search term.

*Nationella patientenkäten, Sverige Landsting och Regioner i Samverkan (2015). Rapport Analysuppdrag: Modellutveckling, utvärdering samt tidigare studier och enkäter. https://skr.se/download/18.40c889381840e60521aa1a14/1668006119029/Rapport%20Analysuppdrag_Modellutveckling,%20utv%C3%A4rdering%20samt%20tidigare%20studier%20och%20enk%C3%A4ter_2015.pdf
