# Supplementary material for: Development of a Person-Centred Coordinated Care Pathway in Swedish Healthcare for Low Back Pain
Source: Int J Integr Care. 2025 May 9;25(2):8. doi: 10.5334/ijic.8940 (PMC12063581; doi:10.5334/ijic.8940)
Supplement: Appendices. — Appendix A–K. [file ijic-25-2-8940-s1.zip › ijic-8940_abbott-s10.pdf]

Appendix J. Example of patient information

Understand and manage my back pain

Back pain

Almost everyone suffers from back pain at some point in their life. Lower back pain is often something that comes and goes or a condition with varying degrees of pain and discomfort. An episode of back pain usually improves within 2-6 weeks even without special treatment. Most people have a mild pattern of discomfort over time that is interrupted by particularly good or bad periods. This back pain is called benign or common back pain. It does not mean that the condition worsens over the years.

Other complaints

In addition to back pain, you may have pain in the buttocks and in one or both legs. You may have difficulties with activities in daily life. This can lead to you experiencing frustration, depression, and anxiety. Some may become afraid to move and become inactive. All of this means that your everyday life can be affected when you experience an episode of back discomfort.

Experience of back pain

Pain arises in the back and is interpreted in the brain. How the pain is interpreted depends on experience, thoughts, feelings, and expectations. This pain system can also become hypersensitive and in some cases, the pain can persist even though what was originally the cause of the pain has healed.

What causes my back pain?

The back is a very strong and stable part of the body and consists of vertebrae, discs, joints, stabilizing ligaments, nerves, and deep and outer muscles. Common back pain is not the same as injury but often comes with temporary irritation or inflammation of one or more of the back's structures at the same time. It is often difficult to clarify exactly which structure is causing the pain. Loading the back unilaterally or too little can be a cause of back pain.

Can medical imaging explain what is causing my back pain?

No - usually the cause of your back pain is not visible on examinations such as X-ray, magnetic camera, or computed tomography. These can only be used if healthcare suspects serious diagnoses such as fracture, cancer, infection, and compression of nerves that control bladder and bowel functions or rheumatic disease. These diseases are rare and are found in fewer than 1 in 100 people with back pain. It is also rare with persistent leg pain where X-ray examination is needed to determine if there is a compression of nerves to the legs if surgery is considered.

It is common for the back to change with age but something that surprises many is that there is no direct correlation between changes in the spine and common back pain. This means that changes in X-ray, magnetic camera, and computed tomography can show pronounced degenerative (age) changes or herniated discs in a completely pain-free person, while someone with pronounced pain has very small X-ray verified changes. Therefore, discomfort is usually not due to degeneration or wear and tear. Herniated discs do not necessarily cause symptoms and often heal naturally.

Why should I not X-ray "for safety's sake"?

Unnecessary X-ray entails high healthcare costs and has risks:

- Exposure of radiation
- Common radiological changes create stress and anxiety, even if the findings are usually unimportant
- X-ray is associated with worse treatment outcomes and an increase in unnecessary surgery

Back health

Good back health is a balance between the back's capacity on one side of the scale and physical/mental challenges on the other as in the picture below. Back pain can occur when there is an imbalance between back capacity compared to physical/mental challenges.

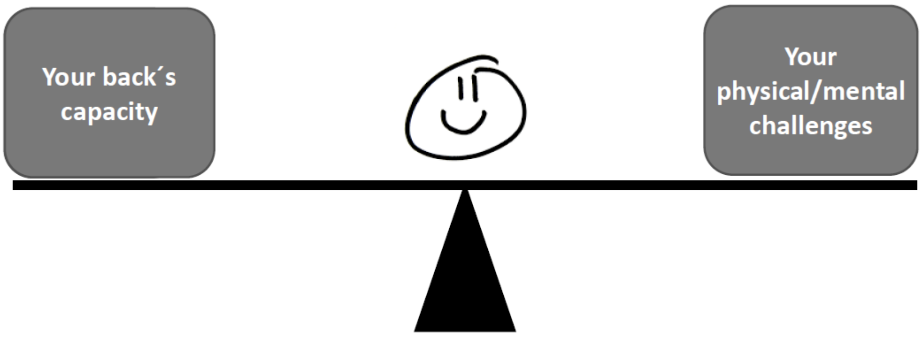

Managing back pain

Advice on self-care

<u>The goal is to get the back's capacity and challenges in balance.</u> You can raise the back's capacity by training strength, flexibility, and improving your general condition. You can adapt your total challenges by modifying the back's physical challenges and mental challenges of everyday stress or negative feelings through a positive attitude and changing your thoughts about your back pain.

The knowledge that the pain comes in periods usually calms. Think about what relieves your discomfort and what you can do yourself that shifts focus from or relieves back pain when you have a bad period. During the acute phase, most people need to take it a little easier and adapt their activities. However, today there is extensive research recommending *not to increase bed rest.* This can delay recovery and reduce confidence in your own ability to be active and deal with your discomfort. *Instead, it is good to return to normal activities as quickly as possible.* Also stay at work or go back as soon as possible, even if you still have back pain.

The most effective way to relieve pain is through some form of physical activity that you feel works to perform at a moderately strenuous / heart-raising intensity for at least 15 minutes duration to induce the secretion of the body's own pain-relieving substances called endorphins. These can provide pain relief effect for up to a day and therefore such a form of physical activity should be performed daily. You can use a pain management scale to be able to find the right load during activities in everyday life and also when you exercise. This model is based on you having an acceptable perceived increased level of pain during an activity and that you are back to the initial pain within 24 hours after the activity. This means that as long as the pain falls back to the starting point within 24 hours, an activity may increase the pain within an acceptable pain level. If you are unsure about your load level, consult your healthcare provider.

General recommendations

- Take it easy the first day/days at the onset of pain if needed. Try to return to normal daily activities as quickly as possible.
- Try gentle exercise such as walking, Nordic walking, swimming, and movements. If you experience pain, you can use the pain management model.
- Consider over-the-counter pain relief medication if needed (Lowest dose and shortest possible treatment time).

Contact your healthcare provider if you have any of the following symptoms in addition to your back pain:

1. Difficulty emptying/controlling bladder and bowel.
2. Reduced sensation in the genitals, around the rectum, or both legs.
3. A feeling of instability when standing.
4. Pain that gets worse instead of better over several weeks.
5. A feeling of illness (e.g., fever or unexplained weight loss)

Nationellt system
för kunskapsstyrning
Hälso- och sjukvård
SVERIGES REGIONER I SAMVERKAN
