# Supplementary material for: Development of a Person-Centred Coordinated Care Pathway in Swedish Healthcare for Low Back Pain
Source: Int J Integr Care. 2025 May 9;25(2):8. doi: 10.5334/ijic.8940 (PMC12063581; doi:10.5334/ijic.8940)
Supplement: Appendices. — Appendix A–K. [file ijic-25-2-8940-s1.zip › ijic-8940_abbott-s11.pdf]

Appendix K. Example of rehabilitation plan/ patient contract

Patient contract – Rehabilitation plan

Summary of assessment findings:

Goals for treatment/intervention:

Primary goal:

Sub-goals:

What can i do by myself (self-management)?

Time plan and scope:

What can the health care service help me with?

Time plan and scope:

Follow-up:

☐ Come back for a follow-up visit____.

☐ Come back for a follow-up visit if your back pain has not improved after ___________ weeks.

☐ As long as your back pain improves you dont need to come back for a follow-up visit.
