# Supplementary material for: Development of a Person-Centred Coordinated Care Pathway in Swedish Healthcare for Low Back Pain
Source: Int J Integr Care. 2025 May 9;25(2):8. doi: 10.5334/ijic.8940 (PMC12063581; doi:10.5334/ijic.8940)
Supplement: Appendices. — Appendix A–K. [file ijic-25-2-8940-s1.zip › ijic-8940_abbott-s2.docx]

Appendix B. Description of interventions in the P3C pathway algorithm

| Healthcare giver interventions | Patient interventions  (to the best of one’s ability) |
| --- | --- |
| ***Track 1: First-line assessment and treatment***  (A) First contact with health care   - Should occur with a licensed healthcare professional, on the same day that the patient has sought contact with the healthcare system. - Can occur in different ways, such as telephone, digital contact through e.g. 1177.se or a physical visit to a healthcare provider. - In line with the recommendations for the P3C pathway for LBP (Appendix C), healthcare professionals should conduct assessment of urgency (Appendix D) and screening of red flags (Appendix E) to assess suspicion of other illness or condition that should receive emergency care.   NOTE: First-line assessment and treatment, steps (A) – (F) can take place at the same time or at different times. | - Describe symptoms, self-care strategies trialed, fears and expectations. |
| (B) Decision: Need for clinical investigation in primary care?   - - Yes: for more severe problems that are suspected to originate from the lumbar spine and are not deemed to require emergency care or mild to moderate problems that have not improved within two to three weeks after the onset – proceed to (C). The investigation should take place within three days of the first contact with the healthcare system.   - No: in the case of mild to moderate symptoms that have lasted less than two to three weeks, give advice on self-care (Appendix J) – proceed to exit the algorithm, the description of interventions in the course of care is concluded.   - No: in case of suspicion of serious illness that meets the criteria for immediate action, the patient should be taken care of in emergency care – proceed to exit the algorithm, the description of interventions in the course of care is concluded. | • Participate in decision-making. |
| (C) Clinical investigation and biopsychosocial assessment   - Within three days of the patient seeking contact, a clinical investigation and biopsychosocial assessment should be carried out with the support of the recommendations and tools of the care pathway (Appendix D-I). This is primarily done by a licensed physiotherapist, alternatively a licensed chiropractor or licensed naprapath depending on the healthcare organisation and includes the following: - Medical history including screening of current complaints, red, yellow and blue flags, and consideration for comorbidities and lifestyle habits (Appendix G). Screening forms should be used at new visits or at an early stage (Appendix D-F). - In patients with a previous history of cancer, there is a risk that new back problems are due to metastasis in the vertebral column even if the patient has been considered cured of their cancer. This is especially true in the case of new pain in the thoracic spine. For patients with previous cancer, MRI (as part of extended cancer investigation) should therefore be considered within 2-3 weeks if there is no improvement in new back problems. In the event of motor impairment, these patients should be investigated urgently. - Physical assessment including general review of systems, neurological and mechanical assessment of spine in line with Appendix G. - Functional and activity assessment. Patient-reported evaluation instruments can be used (Suggestions for instruments, see Appendix H). - Assessment of the patient's pain mechanisms if they can be derived from being nociceptive, neuropathic, nociplastic or mixed forms (Appendix G). - Make a diagnosis based on findings and enter the diagnosis code (Appendix I). | - Describe symptoms, self-care strategies trialled, fears and expectations. - Participate in decision-making. - Ask questions about the reason for and interpretation of tests included in the investigation and the importance of self-care - Involve relatives if necessary |
| (D) Decision: Continued management within the care pathway?   - Yes: for more severe complaints suspected to be of lumbar origin or mild to moderate complaints that have not improved within two to three weeks of onset – proceed to (E). - No: if serious illness is suspected that meets the criteria for immediate action (eg fracture, infection), the patient should be taken care of in emergency care - proceed to exit the algorithm, the description of interventions in the course of care is concluded. - No: if a disease is suspected that does not meet the criteria for immediate action (e.g. inflammatory back diseases, osteoporosis, neurological diseases), refer to specialised care or treatment according to current guidelines - proceed to exit the algorithm, the description of interventions in the course of care is concluded. - No: in case of complaints that improve – proceed to the exit the algorithm, the description of interventions in the course of care is concluded. | - Participate in decision-making. |
| **(E) Information, dialogue and rehabilitation plan**  Healthcare measures Have a dialogue with the patient about diagnosis, prognosis based on identified factors associated with recovery and risk of continued problems, the patient's main goals and sub-goals, and planned treatment as below.  Provide individualised and, where applicable, work-oriented interventions in line with the P3C pathway recommendations (Appendix C). The interventions are carried out by licensed healthcare personnel, preferably in consultation with the employer and the patient.  Basic treatment should consist of the following measures:  • Individualised patient education individually or in groups (for example explanatory model of the patient's symptoms/complaints and pain management strategies) (Appendix J), which can be carried out by all licensed personnel.  • An individually tailored physical training program that can be designed by a physiotherapist, chiropractor or naprapath.  Adjunct treatment means that more interventions are considered in addition to first-line treatment based on the complexity of the patient's problem. If several professions are involved, it is important that they work together as a team. The adjunct treatments may include the following: • Spinal mobilisation and/or manipulation techniques. These treatments can be performed by a physiotherapist, chiropractor or naprapath. • Advice regarding non-prescription analgesics. Doctors can prescribe analgesics if necessary. Clear and time-bound goals should be formulated for pharmacological treatment. Plan for evaluation of treatment and discontinuation of medication. Use the recommendations of the P3C pathway (Appendix C). • Medical certificate for sickness benefit. Use the National Board of Health and Welfare's insurance medical decision support for acute lumbago, lumbago-sciatica or disc herniation. • Rehabilitation interventions to be able to remain in, return to or enter working life can be carried out by occupational therapists, physiotherapists or psychologists. Rehabilitation coordinators can be involved to coordinate between different stakeholders. • Behavioral medicine interventions with a focus on pain and stress management strategies and, if necessary, therapy focusing on improving quality of sleep can be used to reduce the impact of yellow flags on mental well-being. These treatments can be carried out by psychologists, psychotherapists or other licensed health care professionals with the necessary skills. • Time plan regarding scope for implementation of interventions and follow-up. • Establishing or revising a rehabilitation plan - What must happen, when and by whom (Appendix K). | - Participate actively in discussions and design of rehabilitation plans, especially with regard to goal setting and self-care. - Request information if something is unclear. - Involve relatives if necessary. |
| (F) First-line treatment and eventual adjunct treatment   - - Healthcare measures Start treatment according to the rehabilitation plan | - Participate actively in patient education. - Perform physical exercise according to recommendations. - Perform self-care according to recommendations. - Participate actively in any additional treatments. - Involve relatives if necessary. |
| (G) Follow-up och first-line and eventual adjunct treatments   - Healthcare measures Follow-up with the same therapist is preferred. Follow up the outcome of treatment according to (F) in dialogue with the patient about the course of the problem so far, remaining low back problems and current functional and activity ability, prognosis and relapse prevention, goals and rehabilitation plan, adherence to treatment interventions and their effects, sick leave, and patient-reported outcome measures (Appendix H). | - Provide feedback on goal achievement, successes, obstacles, any problems. - Participate actively in the revision of the rehabilitation plan/ patient contract, especially goal setting and self-care. |
| (H) Decision: Need for extended investigation?   - Healthcare measures Yes: if spinal structural pathology is suspected and there is no improvement after a treatment period of evidence-based treatment in primary care - proceed to (J). Yes: patients at high risk of continued complications where multiple yellow flags have been identified - proceed to (J). No: the discomfort is expected to decrease with first-line treatment and any adjunct treatments - continue to (I). | - Participate in decision-making. |
| (I) Decision: Need for continued healthcare?   - Healthcare measures Yes: go back to (D) for a new decision on the patient's continued care. No: the patient can cope with continued self-care - proceed to exit the algorithm, the description of interventions in the course of care is concluded. | - Participate in decision-making. |
| (J) Extended investigation  Healthcare interventions This means that the primary investigation is expanded, and more aspects based on biopsychosocial factors are taken into account, by more professions working together in a multiprofessional team (doctor, physiotherapist, psychologist, nurse, etc.).  In-depth history and clinical status based on Appendix G and includes the following:   - The duration and course of low back pain and possible leg pain and other back-related manifestations. - Symptom description, location, duration. Confirm/deny radiating symptoms in the legs. - Previously completed treatment and its effect. - Relevant previous or current illnesses/injuries/operations that are of importance for surgery. For example, previous back surgery, heart, lung, vascular disease, diabetes, smoking, mental illness, inflammatory diseases and presence of neuropsychiatric conditions. - In-depth examination of yellow and blue flags. - Questions about exposure to violence.   Further investigation regarding possible differential diagnosis. Consider the need for medical imaging, laboratory tests, clinical physiology examinations, and extended psychological investigation. Investigate the following:   - BMI (>32 relative contraindication to spine surgery). - Smoking (6-8 weeks smoke-free before and after surgery). - Neurology: SLR/Random test, sensitivity, motor skills, reflexes.   If the patient has persistent activity-limiting pain problems after a period of evidence-based treatment methods in primary care rehabilitation has been carried out and there is suspicion of spinal structural pathology, investigate further with Magnetic Resonance Tomography (MRI). MRI is the first choice for radiological investigation of low back problems when referred to a spine surgery clinic and should not be older than 12 months. Treatment should have been carried out according to the following time indications, before an MRI investigation is relevant:   - 4–6 weeks of rehabilitation without improvement in severe pain with sciatica, otherwise wait 3–6 months as most spontaneously recover within that time. - Spinal stenosis: After 8–12 weeks with severe pain and reduced ability to walk despite undergoing rehabilitation. - Immediate referral to emergency care if serious pathology is suspected (presence of red warning flags). - Considered at six months with lumbago without sciatica with significant complaints and still unclear genesis.   Other investigations:   - Plain X-ray only if vertebral compression is suspected. - Computed tomography (CT) - only in case of contraindications for MRI. It is important to investigate whether a contraindication really exists. All orthopaedic implants and most pacemakers are MRI compatible. - Neurophysiology - consider in the case of diffuse numbness of lower legs and feet to differentiate nerve root involvement in spinal stenosis compared to polyneuropathy.   Reassess the patient's pain mechanisms, whether they can be deduced to be nociceptive, neuropathic, nociplastic or mixed forms.  In most cases, individualised first-line treatment and eventual adjunct treatment should in most cases continue throughout the assessment process and any waiting time in track 2. | - Participate actively in the investigation. - Ask questions about the reason for and interpretation of measures included in the investigation. - Involve relatives if necessary. |
| (K) Decision: Spinal pathology with indication for surgery?  Healthcare interventions Yes: if the patient's symptoms and MRI findings indicate surgery, there are no contraindications for back surgery, and the patient is motivated to undergo back surgery, make a diagnosis based on findings and enter the diagnosis code (Appendix I). Continue to (L). No: continue to (N). | - Participate in decision-making. - Ask questions about the reason for and interpretation of measures included in the investigation. |
| (L) Referral for spinal surgeon assessment   - Healthcare measures Referral includes information about investigations and treatments that have been carried out. |  |
| (M) Decision: Referral reply – Spinal surgery?  Healthcare interventions Yes: While waiting for back surgery, revise rehabilitation plan/ patient contract with prehabilitation interventions in the form of physical training aimed at promoting physical activity level and ADL. Prehabilitation takes place before the patient begins a surgical treatment and is about giving the patient the best conditions to cope with the planned surgical treatment. If back surgery treatment has started, proceed to exit the algorithm, the description of interventions in the course of care is concluded. No: continue to (N). | - Participate in decision-making. - Actively participate in the revision of the rehabilitation plan/ patient contract, especially goal setting and self-care. |
| (N) Decision: Transition to the care pathway for chronic pain?  Healthcare interventions  Does the patient have complex long-term pain problems and is deemed unable to be handled further in this course of care? Yes: Revise the rehabilitation plan, proceed to exit the algorithm, the description of interventions in the course of care is concluded. No: Return to (D). | - Participate in decision-making. - Actively participate in the revision of the rehabilitation plan/ patient contract, especially goal setting and self-care. |
