# Supplementary material for: Development of a Person-Centred Coordinated Care Pathway in Swedish Healthcare for Low Back Pain
Source: Int J Integr Care. 2025 May 9;25(2):8. doi: 10.5334/ijic.8940 (PMC12063581; doi:10.5334/ijic.8940)
Supplement: Appendices. — Appendix A–K. [file ijic-25-2-8940-s1.zip › ijic-8940_abbott-s3.docx]

Appendix C. National Work Group's Basis for Recommendations on Assessment and Treatment of Low Back Pain

The recommendations have been developed by the National Work Group in a consensus process and review of systematic reviews and can be seen as an expert opinion that needs to be updated and revised in the future. The recommendations and their available evidence are at the group level and should be seen as a support but does not replace clinical assessment. Examination and treatment must always be individually adapted.

|  | **The recommendations of the P3C pathway are based on the combination of four perspectives** | | | |
| --- | --- | --- | --- | --- |
|  | **Balance between wanted and unwanted effects** | **Scientific evidence** | | **Values and preferences** |
|  |  | **Effectiveness** | **Costs** |  |
| **Clinical investigation and biopsychosocial assessment** |  |  |  |  |
| 1. Individualisation of standardised processes should be used for medical history and physical assessment, preliminary prognosis and diagnosis, including thorough screening to exclude severe pathology. | Desired effect:  Early detection  of serious  pathology.  The patient receives prognostic information.  Undesired effect:  False positive  or false  negative  results of  screening can occur  despite adequate reliability. | Best available evidence supports the recommendation  for standardised investigative methods. A patient-centered clinical assessment is needed to plan individualised interventions and exclude unusual but serious medical conditions (⊗○○○) [1-9]. | Potential for  reduced costs  but reliable  research is lacking  (⊗○○○) [2]. | Standardized processes for similar patients that are at the same time individualisable to patient needs are important for the patient-centered, equitable and quality-assured care. Patients express a strong desire for clear, consistent and individualised information on  prognosis [10]. |
| 2. Imaging (X-ray, CT, MRI) should only be used with clear indication. Indications:  1) In case of suspicion of  severe pathology (see red  flags Appendix B and C),  (2) In case of persistent activity limiting pain/discomfort after an adequate period of  evidence-based treatment in  primary healthcare has been  carried out together with  with suspicion of structural spinal pathology | Desired effect:  Prevent  unnecessary use of medical imaging.  Undesirable effect:  Radiation. | Unnecessary use of medical imaging is common. There is only a weak correlation between patient's symptoms and degenerative findings in medical imaging. In addition, degenerative findings are also prevalent in asymptomatic individuals. Patient outcomes do not differ between those who may or may not receive early imaging for benign low back pain (⊗⊗○○) [11-14]. | Medical imaging is  associated with  higher medical costs, increased  healthcare consumption  and more  work absenteeism  (⊗⊗○○) [15]. | There is an overconfidence that medical imaging can confirm the source of the back pain. This can potentially contribute to expectations and  overuse of medical  imaging [10). |
| 3. Screening for psychosocial  Risk factors (yellow flags)  with patient-reported tools (e.g. Örebro Musculoskeletal Pain  Screening Questionnaire (ÖMPSQ) – short version, or  Start Back Screening tool (SBT)) should be used at initial consultation or at an early stage  to investigate the risk of persisting back problems and  to support treatment choices.  E.g. For patients who  classify as 'moderately risk' according to a screening procedure should receive multimodal interventions including physical as well as psychological interventions  and with a focus on good  function and work ability in order to prevent continued/long-term problems. | Desired effect:  Provides valuable information  that helps choose the right level  of treatment  Undesirable effect:  None known. | Currently there is no tool that with good evidence that can predict the risk of developing persistent pain [16]. However, there is evidence for ÖMPSQ and SBT for prognosing disability and return to work and can contribute to better outcome [17-19]. Outcome of the screening is used partly to individualise the content of interventions as well as to inform the scope of treatment (e.g. unimodal or multimodal therapy) (⊗○○○) [19]. | Choice and scope of intervention stratified according to assessment of a risk profile for persistent back pain can give lower  costs compared to  no stratification  (⊗○○○) [19]. | Patients express a strong desire for clear, consistent and individualised information on  prognosis, and treatment options [10]. The use of the tools can possibly provide a better basis for this. According to the work group, the tools should be used to support but not replace the usual history taking and physical examination |
| **First-line treatments** |  |  |  |  |
| 1. Advice on good prognosis,  maintenance of appropriate physical activity and advice against appropriate bed rest should be used as a part of the first-line treatment. | Desired effect:  Maintain adequate function and  activity.  Undesirable effect:  None known. | For acute and persistent LBP the  literature shows a statistically significant benefit for advice on normal activity compared to  advice on bed rest for pain intensity (⊗⊗○○) and function (⊗⊗⊗○). Evidence on  whether the improvement is so great that it's clinically relevant is missing [20]. | Interventions that include an "information and advice” component can lead to a reduction in work  absenteeism (⊗○○○) [21]. | Patients express a  strong desire for clear, consistent and  individualized information on prognosis [10].  The work group considers that appropriate physical activity is likely to contribute to maintenance of patients’ function and wellbeing. |
| 2. Individualized patient education should be used as part of first-line treatment  (e.g. explanatory model of  patient's symptoms/complaints). | Desired effect:  Increase patient's understanding of pain and ability to manage pain.  Undesirable effect:  None known. | Patient education about pain and on pain management focused on thoughts, feelings och behaviour combined with exercise therapy may reduce pain in the future and enhance function. For patients with persistent low back pain a clinically relevant improvement regarding pain and function may occur compared to no intervention or minimal intervention (⊗⊗○○) [22–24]. | Patient education may be cost effective, particularly in combination with exercise therapy  (⊗○○○) [21]. | Patients express a desire for support in how to prevent or manage existing and future barriers regarding low back pain related to function and ability to work [10].  The work group considers that individualized patient education in existing context is optimal. Health care professionals should manage to deliver unanimous information during the process. |
| 3. Individualized exercise therapy should be used as part of first-line treatment for patients in need. | Desired effect:  Reduction in experience of pain. Maintain or improve the level of function and activity.  Undesirable effect:  Mild temporary soreness in lower back. | Exercise therapy for chronic low back pain results in clinically relevant reduction in pain compared to no exercise therapy, usual care, or placebo. For acute LBP no such difference is evident. There is not enough evidence of clinically relevant differences for one exercise therapy compared to another. Nor that supervised exercise therapy would be superior to unsupervised. Support exists for an increased dosage to improve the effect. Treatment periods usually range from six to twelve weeks with a frequency of one to two times a week. (⊗⊗⊗○) [25-28]. | Exercise therapy may be cost effective, particularly in combination with patient education (⊗○○○) [21,28]. | Individualised exercise therapy programs are preferred by patients [10].  The work group recommends that appropriate exercise therapy is individualized to the patients' preferences, goals, comorbidities and current complexity. Supervised exercise therapy should always strive for independent performance. This should be enhanced with a prescription of physical activity with a follow-up. |
| **Non-pharmacological adjunct therapies if first-line treatments are not sufficiently effective** |  |  |  |  |
| 1. Manual mobilization and/or manipulation techniques on the lumbar spine can be trialed on the right indication as an addition to first-line treatment. | Desired effect:  Short-term analgesic effect. Maintaining/improving the patient's level of function and activity.  Undesirable effect:  Symptom increase (Usually temporary and mild, but rare serious effects may occur with manipulation techniques). | Mobilizing and/or manipulation techniques for acute low back pain have not been shown to be more effective than sham treatment but may provide a slight improvement in pain and function in the short term beyond basic treatments. Similar evidence exists for long-term low back pain, but in the short term, effects on pain intensity are shown in addition to sham treatment. However, it is uncertain whether the effect is large enough for patients to experience a clear improvement over time. At the group level, there is no evidence that one technique would be superior to another (⊗⊗⊗○) [29-32]. | The treatment method can be cost-effective, when done in addition to patient education and physical exercise. A typical period of treatment described in research studies consists of 4–8 treatments over 4–8 weeks (⊗○○○) [21]. | The effects of short trial treatments should be evaluated before deciding on a complete treatment period. The work group believes that overuse of passive therapist-dependent adjunctive therapies can reduce active interventions and worsen outcomes. |
| 2. Acupuncture can be trialed, if necessary, as an adjunct to first-line treatment. | Desired effect:  Short-term analgesic effect. Maintaining/improving the patient's level of function and activity.  Undesirable effect:  Symptom increase (though usually temporary and mild). | Acupuncture for acute low back pain has been shown to be more effective than sham or NSAIDs for small but not always clinically relevant short-term reduction of pain and drug use (⊗⊗○○) [33-34]. Studies on chronic low back pain show that acupuncture is more effective than sham treatment, no treatment and, in addition to basic treatments, for small but not always clinically relevant short-term reduction of pain and function (⊗⊗○○) [33-39]. | It cannot be ruled out that acupuncture may be cost-effective for certain subgroups of patients, but more studies are needed (⊗○○○) [21]. | The effects of short trial treatments should be evaluated before deciding on a complete treatment period. The working group believes that overuse of passive therapist-dependent adjunctive therapies can reduce active interventions and worsen outcomes. |
| 3. Transcutaneous electrical nerve stimulation (TENS) can be trialed, if necessary, as an adjunct to first-line treatment. | Desired effect:  Short-term analgesic effect. Maintaining/improving the patient's level of function and activity.  Undesirable effect:  Symptom increase (though usually temporary and mild). | TENS for acute low back pain has been shown to be more effective than short-term sham treatment in conditions with moderate-high pain intensity (⊗○○○) [40]. TENS for chronic low back pain has not been shown to be more effective than sham treatment for pain intensity but may provide small but not always clinically relevant short-term improvement in function (⊗⊗○○) [41]. | There is a lack of economic analyses on low back pain. | The effects of short trial treatments should be evaluated before deciding on a complete treatment period. The working group believes that overuse of passive adjunctive therapies can reduce active measures and worsen outcomes. |
| 4. Mechanical lumbar traction ***should not be routinely used*** as an adjunct to first-line treatment for low back pain ***but may be considered for radicular leg pain*** as an adjunct to first-line treatment. | Desired effect:  Short-term analgesic effect. Maintaining/improving the patient's level of function and activity.  Undesirable effect:  Symptom increase (though usually temporary and mild). | Mechanical traction for acute or chronic low back pain has not been shown to be more effective than sham or no treatment or in addition to first-line treatments (⊗⊗⊗○) [42]. Supine mechanical traction of the lumbar spine for radicular leg pain has shown small short-term effects beyond basic treatment for pain intensity and function. However, it is uncertain whether the effect is large enough for patients to experience a clear improvement (⊗⊗○○) [43,44] | There is a lack of economic analyses on low back pain. | The effects of short trial treatments should be evaluated before deciding on a complete treatment period. The working group believes that overuse of passive adjunctive therapies can reduce active measures and worsen outcomes. |
| 5. Corsets, shoe inserts/insoles and ultrasound, ***should not be routinely used*** as an adjunct to first-line treatment. | Desired effect:  Prevent the routine use of interventions that do not have scientific support for effectiveness.  Undesirable effect:  Individual assessment is not made and those who could benefit from the intervention will be left without this treatment. | Treatments such as corset (⊗○○○) [45], shoe inserts/insoles (⊗○○○) [46], and ultrasound (⊗⊗○○) [47], have been shown to be no more effective than sham therapy or no treatment and have no clinically relevant effects beyond other therapeutic measures. | There is a lack of economic analyses on low back pain. | There may be overconfidence that these treatments can improve LBP. This can potentially contribute to expectations and overuse of these interventions. It cannot be excluded that subgroups of patients may experience positive effects of these measures when the intended mechanism of effect is targeted at specific disabilities. |
| **Pharmacological adjunct therapies if first-line treatments are not sufficiently effective** |  |  |  |  |
| 1. COX inhibitors may be used as necessary as an adjunct to basic treatment (lowest effective dose, shortest duration of treatment, regular evaluation of efficacy and side effects. Treatment should be discontinued in the absence of efficacy. | **Desired effect:**  Analgesic effect (Painlessness is rarely the goal).  **Undesirable effect:**  Increased risk of gastrointestinal upset and cardiovascular risk in the long term. Risk of routine prescribing without evaluation of efficacy. | There is evidence of significant effects of non-steroidal anti-inflammatory drugs (NSAIDs) in addition to placebo effects for patients with acute or prolonged low back pain in terms of pain and function (⊗⊗⊗○) [48]. However, the effects are small and not always clinically relevant (⊗⊗⊗⊗) [49-50]. Long-term follow-up of effects is lacking. | NSAIDs may be more cost-effective than other drugs (⊗○○○) [51]. | The patient's cardiovascular risk and renal function should be considered. COX inhibitors are preferred over non-selective NSAIDs for minimizing gastrointestinal upset. |
| 2. Paracetamol **should not be routinely used for acute low back pain** as an adjunct to basic treatment. | **Desired effect:**  Prevent routine use.  **Undesirable effect:**  An individual assessment is not carried out and those who could benefit from the treatment are left without it. | Repeated doses of paracetamol have no effect beyond placebo on pain and function reported by patients with acute low back pain (⊗⊗⊗⊗) [52–54]. There is a lack of research evaluating the effect of paracetamol on subacute or long-term low back pain. There are no significant differences in side effects beyond placebo, but dose-dependent side effects from paracetamol in the form of dose-dependent liver effects are known. | Paracetamol for acute low back pain leads to increased health care costs and can lead to increased disability, longer duration of symptoms and absenteeism [55]. | The working group is of the opinion that since the risks of the treatment are so small, it can be considered in selected cases at the individual level, but then with careful evaluation of the effect and that the treatment is discontinued if the desired effect is not achieved, in order to prevent routine long-term prescribing. Future research in patients with subacute or long-term low back pain is needed to clarify the uncertainty surrounding effects [52]. |
| 3. Opioids should not be routinely used as an adjunct to basic treatment. | **Desired effect:**  Prevent routine use.  **Undesirable effect:**  Individual assessment is not made and those who could benefit from the treatment are left without it. | For acute and long-term low back pain with or without leg pain, opioids have been shown to be more effective than placebo but **not have a better effect on pain and function than other analgesics such as NSAIDs**.  A broad and more severe spectrum of adverse reactions to opioids exists compared to other analgesics (⊗⊗○○) [56–61]. | Opioids for low back pain led to more frequent and long-term analgesic use and disability compared to other analgesics (⊗○○○) [51,55]. | The working group believes that the treatment carries more risks than benefits for most patients. Any treatment trials at the individual level should be preceded by a careful risk assessment for iatrogenic dependence, the lowest effective doses should be used, treatment should be regularly monitored for effects, side effects and signs of tolerance development, and should be discontinued when the risks outweigh the benefits [62]. |
| 4. Antiepileptic medicinal products (gabapentinoids) **should not be routinely used** as a first-line adjunct to basic treatment. | **Desired effect:**  Prevent routine use.  **Undesirable effect:**  Individual assessment is not made and those who could benefit from the treatment are left without it. | Gabapentinoids have no effect beyond placebo for acute and prolonged low back pain, sciatica pain or function. In addition, there is a significantly increased risk of adverse reactions compared to placebo (⊗⊗⊗⊗) [63–65]. | There is a lack of economic analyses of low back pain. | The working group considers that the treatment entails greater risks than benefits for patients with low back disorder and is likely to lead to increased direct and indirect costs. Treatment is common for neuropathic pain, but antidepressants should be tried first. |
| 5. Antidepressants (TCA SNRIs) for radicular leg pain can be used as needed as an adjunct to basic treatment (lowest effective dose, shortest duration of treatment, regular evaluation of efficacy and side effects. Treatment should be discontinued in the absence of efficacy. | **Desired effect:**  Analgesic effect (it is rarely painlessness that is the goal).  **Undesirable effect:**  Increased risk of mild side effects. | Serotonin and norepinephrine reuptake inhibitors have small but not always clinically relevant effects beyond placebo for low back pain and function (⊗⊗⊗○). In radicular leg pain (neuropathic component), tricyclic antidepressants, serotonin and norepinephrine reuptake inhibitors have clinically relevant analgesic efficacy in addition to placebo (⊗○○○).  Antidepressants do not carry an increased risk of serious side effects but a slight increase in mild side effects, such as nausea, beyond placebo (⊗○○○) [66-68]. | Antidepressants may be more cost-effective than other drugs except NSAIDs (⊗○○○) [51,55].  In subgroups at risk of NSAID-related adverse events, antidepressants are particularly cost-effective. | The working group considers that treatment may be considered if the use of COX inhibitors is inappropriate or does not produce the desired effect in low back pain. |
| 6. Muscle relaxants for acute low back pain may be used as necessary as an adjunct to basic treatment (lowest effective dose, shortest duration of treatment, regular evaluation of efficacy and side effects). Treatment should be discontinued in the absence of efficacy. | **Desired effect:**  Analgesic effect (it is rarely painlessness that is the goal).  **Undesirable effect:**  Increased risk of mild side effects. | Muscle relaxants have small but not always clinically relevant effects beyond placebo for acute low back pain. Muscle relaxants do not increase the risk of serious adverse reactions, but a slight increase in mild adverse reactions beyond placebo is seen (⊗○○○) [69]. | There is a lack of economic analyses of low back pain. | The working group believes that the treatment may be relevant in severe acute low back pain. |
| **Referral to specialized care for benign complex conditions** |  |  |  |  |
| 1. Referral to specialized care should be considered if there is no improvement after an adequate period of evidence-based treatment methods in primary care.  - In case of continued discomfort/untenable pain situation and MRI-verified back pathology; referral to an orthopedic clinic.  - In case of complex long-term pain conditions; referral to a pain clinic. | Desired effect:  Referral to specialized care for the right indication and at the right time.  Unwanted effect:  None identified | There is a lack of randomized clinical studies that analyze the effectiveness of these processes in the treatment of low back problems | There is a lack of economic analyses on low back problems | The work group believes these processes can improve the selection of the right patients who should be referred to specialized care and reduce unnecessary referrals. |

Reported evidence strength has been taken from the studies referred to. Overall strength of evidence: Strong scientific basis (⊗⊗⊗⊗), Moderate scientific basis (⊗⊗⊗○), Limited scientific basis (⊗⊗○○), Insufficient scientific basis (⊗○○○).

References

1. Coeckelberghs E, Verbeke H, Desomer A, Jonckheer P, Fourney D, Willems P, Coppes M, Rampersaud R, van Hooff M, van den Eede E, Kulik G, de Goumoëns P, Vanhaecht K, Depreitere B. International comparative study of low back pain care pathways and analysis of key interventions. Eur Spine J. 2021 Apr;30(4):1043-1052.
2. May S, Runge N, Aina A. Centralization and directional preference: An updated systematic review with synthesis of previous evidence. Musculoskelet Sci Pract. 2018 Dec;38:53-62.
3. Maselli F, Palladino M, Barbari V, Storari L, Rossettini G, Testa M. The diagnostic value of Red Flags in thoracolumbar pain: a systematic review. Disabil Rehabil. 2022 Apr;44(8):1190-1206.
4. Fernando SM, Tran A, Cheng W, Rochwerg B, Strauss SA, Mutter E, McIsaac DI, Kyeremanteng K, Kubelik D, Jetty P, Nagpal SK, Thiruganasambandamoorthy V, Roberts DJ, Perry JJ. Accuracy of presenting symptoms, physical examination, and imaging for diagnosis of ruptured abdominal aortic aneurysm: Systematic review and meta-analysis. Acad Emerg Med. 2022 Apr;29(4):486-496.
5. Dionne N, Adefolarin A, Kunzelman D, Trehan N, Finucane L, Levesque L, Walton DM, Sadi J. What is the diagnostic accuracy of red flags related to cauda equina syndrome (CES), when compared to Magnetic Resonance Imaging (MRI)? A systematic review. Musculoskelet Sci Pract. 2019 Jul;42:125-133.
6. Henschke N, Maher CG, Ostelo RW, de Vet HC, Macaskill P, Irwig L. Red flags to screen for malignancy in patients with low back pain. Cochrane Database Syst Rev 2013; 2: CD008686.
7. Yusuf M, Finucane L, Selfe J. Red flags for the early detection of spinal infection in back pain patients. BMC Musculoskelet Disord. 2019 Dec 13;20(1):606.
8. Williams CM, Henschke N, Maher CG, van Tulder MW, Koes BW, Macaskill P, Irwig L. Red flags to screen for vertebral fracture in patients presenting with low-back pain. Cochrane Database Syst Rev 2013; 1: CD008643.
9. Sepriano A, Rubio R, Ramiro S, Landewé R, van der Heijde D. Performance of the ASAS classification criteria for axial and peripheral spondyloarthritis: a systematic literature review and meta-analysis. Ann Rheum Dis. 2017 May;76(5):886-890.
10. Lim YZ, Chou L, Au RT, Seneviwickrama KMD, Cicuttini FM, Briggs AM, Sullivan K, Urquhart DM, Wluka AE. People with low back pain want clear, consistent and personalised information on prognosis, treatment options and self-management strategies: a systematic review. J Physiother. 2019 Jul;65(3):124-135.
11. Brinjikji W, Luetmer PH, Comstock B, Bresnahan BW, Chen LE, Deyo RA, Halabi S, Turner JA, Avins AL, James K, Wald JT, Kallmes DF, Jarvik JG. Systematic literature review of imaging features of spinal degeneration in asymptomatic populations. AJNR Am J Neuroradiol. 2015 Apr;36(4):811-6.
12. Raastad J, Reiman M, Coeytaux R, Ledbetter L, Goode AP. The association between lumbar spine radiographic features and low back pain: a systematic review and meta-analysis. Semin Arthritis Rheum. 2015 Apr;44(5):571-585.
13. Chou R, Fu R, Carrino JA, Deyo RA. Imaging strategies for low-back pain: systematic review and meta-analysis. Lancet. 2009 Feb 7;373(9662):463-72.
14. Jenkins HJ, Downie AS, Maher CG, Moloney NA, Magnussen JS, Hancock MJ. Imaging for low back pain: is clinical use consistent with guidelines? A systematic review and meta-analysis. Spine J. 2018 Dec;18(12):2266-2277.
15. Lemmers GPG, van Lankveld W, Westert GP, van der Wees PJ, Staal JB. Imaging versus no imaging for low back pain: a systematic review, measuring costs, healthcare utilization and absence from work. Eur Spine J. 2019 May;28(5):937-950.
16. SBU. Identifiering av patienter i primärvård som riskerar förvärrad smärta [Internet]. Stockholm: Statens beredning för medicinsk och social utvärdering (SBU); 2022. SBU:s upplysningstjänst. [2022-09-22]. Available from: <https://www.sbu.se/ut202215>.
17. Forsbrand MH, Grahn B, Hill JC, Petersson IF, Post Sennehed C, Stigmar K. Can the STarT Back Tool predict health-related quality of life and work ability after an acute/subacute episode with back or neck pain? A psychometric validation study in primary care. BMJ Open. 2018 Dec 22;8(12):e021748.
18. Karran EL, McAuley JH, Traeger AC, Hillier SL, Grabherr L, Russek LN, Moseley GL. Can screening instruments accurately determine poor outcome risk in adults with recent onset low back pain? A systematic review and meta-analysis. BMC Med. 2017 Jan 19;15(1):13.
19. Ogbeivor C, Elsabbagh L. Management approach combining prognostic screening and targeted treatment for patients with low back pain compared with standard physiotherapy: A systematic review & meta-analysis. Musculoskeletal Care. 2021 Dec;19(4):436-456.
20. Jones CMP, Shaheed CA, Ferreira GE, Kharel P, Lin C-WC, Maher CG. Advice and education provide small short-term improvements in pain and disability in people with non-specific spinal pain: a systematic review. Journal of Physiotherapy. 2021;67:263–270.
21. Andronis L, Kinghorn P, Qiao S, Whitehurst DG, Durrell S, McLeod H. Cost-Effectiveness of Non-Invasive and Non-Pharmacological Interventions for Low Back Pain: a Systematic Literature Review. Appl Health Econ Health Policy. 2017 Apr;15(2):173-201.
22. Ho EK, Chen L, Simic M, Ashton-James CE, Comachio J, Wang DXM, Hayden JA, Ferreira ML, Ferreira PH. Psychological interventions for chronic, non-specific low back pain: systematic review with network meta-analysis. BMJ. 2022 Mar 30;376:e067718.
23. de Campos TF, Maher CG, Fuller JT, Steffens D, Attwell S, Hancock MJ. Prevention strategies to reduce future impact of low back pain: a systematic review and meta-analysis. Br J Sports Med. 2021 May;55(9):468-476.
24. Rathnayake APS, Sparkes V, Sheeran L. What is the effect of low back pain self-management interventions with exercise components added? A systematic review with meta-analysis. Musculoskelet Sci Pract. 2021 Dec;56:102469.
25. Hayden JA, Ellis J, Ogilvie R, Malmivaara A, van Tulder MW. Exercise therapy for chronic low back pain. Cochrane Database Syst Rev. 2021 Sep 28;9(9):CD009790.
26. Hayden JA, Ellis J, Ogilvie R, Stewart SA, Bagg MK, Stanojevic S, Yamato TP, Saragiotto BT. Some types of exercise are more effective than others in people with chronic low back pain: a network meta-analysis. J Physiother. 2021 Oct;67(4):252-262.
27. Karlsson M, Bergenheim A, Larsson MEH, Nordeman L, van Tulder M, Bernhardsson S. Effects of exercise therapy in patients with acute low back pain: a systematic review of systematic reviews. Syst Rev. 2020 Aug 14;9(1):182.
28. Miyamoto GC, Lin CC, Cabral CMN, van Dongen JM, van Tulder MW. Cost-effectiveness of exercise therapy in the treatment of non-specific neck pain and low back pain: a systematic review with meta-analysis. Br J Sports Med. 2019 Feb;53(3):172-181.
29. Rubinstein SM, de Zoete A, van Middelkoop M, Assendelft WJJ, de Boer MR, van Tulder MW. Benefits and harms of spinal manipulative therapy for the treatment of chronic low back pain: systematic review and meta-analysis of randomised controlled trials. BMJ. 2019 Mar 13;364:l689.
30. de Zoete A, Rubinstein SM, de Boer MR, Ostelo R, Underwood M, Hayden JA, Buffart LM, van Tulder MW; International IPD-SMT group: The effect of spinal manipulative therapy on pain relief and function in patients with chronic low back pain: an individual participant data meta-analysis. Physiotherapy. 2021 Sep;112:121-134.
31. Rubinstein SM, Terwee CB, Assendelft WJ, de Boer MR, van Tulder MW. Spinal manipulative therapy for acute low-back pain. Cochrane Database Syst Rev. 2012 Sep 12;2012(9):CD008880.
32. Paige NM, Miake-Lye IM, Booth MS, Beroes JM, Mardian AS, Dougherty P, Branson R, Tang B, Morton SC, Shekelle PG. Association of Spinal Manipulative Therapy With Clinical Benefit and Harm for Acute Low Back Pain: Systematic Review and Meta-analysis. JAMA. 2017 Apr 11;317(14):1451-1460.
33. Su X, Qian H, Chen B, Fan W, Xu D, Tang C, Lu L. Acupuncture for acute low back pain: a systematic review and meta-analysis. Ann Palliat Med. 2021 Apr;10(4):3924-3936.
34. Xiang Y, He JY, Tian HH, Cao BY, Li R. Evidence of efficacy of acupuncture in the management of low back pain: a systematic review and meta-analysis of randomised placebo- or sham-controlled trials. Acupunct Med. 2020 Feb;38(1):15-24.
35. Baroncini A, Maffulli N, Eschweiler J, Molsberger F, Klimuch A, Migliorini F. Acupuncture in chronic aspecific low back pain: a Bayesian network meta-analysis. J Orthop Surg Res. 2022 Jun 20;17(1):319.
36. Asano H, Plonka D, Weeger J. Effectiveness of Acupuncture for Nonspecific Chronic Low Back Pain: A Systematic Review and Meta-Analysis. Med Acupunct. 2022 Apr 1;34(2):96-106.
37. Huang JF, Zheng XQ, Chen D, Lin JL, Zhou WX, Wang H, Qin Z, Wu AM. Can Acupuncture Improve Chronic Spinal Pain? A Systematic Review and Meta-Analysis. Global Spine J. 2021 Oct;11(8):1248-1265.
38. Li YX, Yuan SE, Jiang JQ, Li H, Wang YJ. Systematic review and meta-analysis of effects of acupuncture on pain and function in non-specific low back pain. Acupunct Med. 2020 Aug;38(4):235-243.
39. Mu J, Furlan AD, Lam WY, Hsu MY, Ning Z, Lao L. Acupuncture for chronic nonspecific low back pain. Cochrane Database Syst Rev. 2020 Dec 11;12(12):CD013814.
40. Binny J, Joshua Wong NL, Garga S, Lin CC, Maher CG, McLachlan AJ, Traeger AC, Machado GC, Shaheed CA. Transcutaneous electric nerve stimulation (TENS) for acute low back pain: systematic review. Scand J Pain. 2019 Apr 24;19(2):225-233.
41. Wu LC, Weng PW, Chen CH, Huang YY, Tsuang YH, Chiang CJ. Literature Review and Meta-Analysis of Transcutaneous Electrical Nerve Stimulation in Treating Chronic Back Pain. Reg Anesth Pain Med. 2018 May;43(4):425-433.
42. Wegner I, Widyahening IS, van Tulder MW, Blomberg SE, de Vet HC, Brønfort G, Bouter LM, van der Heijden GJ. Traction for low-back pain with or without sciatica. Cochrane Database Syst Rev. 2013 Aug 19;2013(8):CD003010.
43. Vanti C, Panizzolo A, Turone L, Guccione AA, Violante FS, Pillastrini P, Bertozzi L. Effectiveness of Mechanical Traction for Lumbar Radiculopathy: A Systematic Review and Meta-Analysis. Phys Ther. 2021 Mar 3;101(3):pzaa231. doi: 10.1093/ptj/pzaa231.
44. Cheng YH, Hsu CY, Lin YN. The effect of mechanical traction on low back pain in patients with herniated intervertebral disks: a systemic review and meta-analysis. Clin Rehabil. 2020 Jan;34(1):13-22.
45. Gignoux P, Lanhers C, Dutheil F, Boutevillain L, Pereira B, Coudeyre E. Non-rigid lumbar supports for the management of non-specific low back pain: A literature review and meta-analysis. Ann Phys Rehabil Med. 2022 Jan;65(1):101406.
46. Kong L, Zhou X, Huang Q, Zhu Q, Zheng Y, Tang C, Li JX, Fang M. The effects of shoes and insoles for low back pain: a systematic review and meta-analysis of randomized controlled trials. Res Sports Med. 2020 Oct-Dec;28(4):572-587.
47. Ebadi S, Henschke N, Forogh B, Nakhostin Ansari N, van Tulder MW, Babaei-Ghazani A, Fallah E. Therapeutic ultrasound for chronic low back pain. Cochrane Database Syst Rev. 2020 Jul 5;7(7):CD009169.
48. Machado GC, Maher CG, Ferreira PH, Day RO, Pinheiro MB, Ferreira ML. Non-steroidal anti-inflammatory drugs for spinal pain: a systematic review and meta-analysis. Ann Rheum Dis. 2017 Jul;76(7):1269-1278.
49. van der Gaag WH, Roelofs PD, Enthoven WT, van Tulder MW, Koes BW. Non-steroidal anti-inflammatory drugs for acute low back pain. Cochrane Database Syst Rev. 2020 Apr 16;4(4):CD013581.
50. Enthoven WT, Roelofs PD, Deyo RA, van Tulder MW, Koes BW. Non-steroidal anti-inflammatory drugs for chronic low back pain. Cochrane Database Syst Rev. 2016 Feb 10;2(2):CD012087.
51. Shah D, Anupindi VR, Vaidya V. Pharmacoeconomic Analysis of Pain Medications Used to Treat Adult Patients with Chronic Back Pain in the United States. J Pain Palliat Care Pharmacother. 2016 Dec;30(4):300-307.
52. Saragiotto BT, Machado GC, Ferreira ML, Pinheiro MB, Abdel Shaheed C, Maher CG. Paracetamol for low back pain. Cochrane Database Syst Rev. 2016 Jun 7;2016(6):CD012230.
53. Schreijenberg M, Lin CC, Mclachlan AJ, Williams CM, Kamper SJ, Koes BW, Maher CG, Billot L. Paracetamol is ineffective for acute low back pain even for patients who comply with treatment: complier average causal effect analysis of a randomized controlled trial. Pain. 2019 Dec;160(12):2848-2854.
54. Abdel Shaheed C, Ferreira GE, Dmitritchenko A, McLachlan AJ, Day RO, Saragiotto B, Lin C, Langendyk V, Stanaway F, Latimer J, Kamper S, McLachlan H, Ahedi H, Maher CG. The efficacy and safety of paracetamol for pain relief: an overview of systematic reviews. Med J Aust. 2021 Apr;214(7):324-331.
55. Lin CC, Li Q, Williams CM, Maher CG, Day RO, Hancock MJ, Latimer J, Mclachlan AJ, Jan S. The economic burden of guideline-recommended first line care for acute low back pain. Eur Spine J. 2018 Jan;27(1):109-116.
56. Videman T, Heikkilä J, Partanen T. Double-blind parallel study of meptazinol versus diflunisal in the treatment of lumbago. Curr Med Res Opin. 1984;9(4):246-52.
57. Innes GD, Croskerry P, Worthington J, Beveridge R, Jones D. Ketorolac versus acetaminophen-codeine in the emergency department treatment of acute low back pain. J Emerg Med. 1998 Jul-Aug;16(4):549-56.
58. Plapler PG, Scheinberg MA, Ecclissato Cda C, Bocchi de Oliveira MF, Amazonas RB. Double-blind, randomized, double-dummy clinical trial comparing the efficacy of ketorolac trometamol and naproxen for acute low back pain. Drug Des Devel Ther. 2016 Jun 17;10:1987-93.
59. Sanger N, Bhatt M, Singhal N, Ramsden K, Baptist-Mohseni N, Panesar B, Shahid H, Hillmer A, D Elia A, Luo C, Rogers V, Arunan A, Baker-Beal L, Haber S, Henni J, Puckering M, Sun S, Ng K, Sanger S, Mouravaska N, Samaan MC, de Souza R, Thabane L, Samaan Z. Adverse Outcomes Associated with Prescription Opioids for Acute Low Back Pain: A Systematic Review and Meta-Analysis. Pain Physician. 2019 Mar;22(2):119-138.
60. Nury E, Schmucker C, Nagavci B, Motschall E, Nitschke K, Schulte E, Wegwarth O, Meerpohl JJ. Efficacy and safety of strong opioids for chronic noncancer pain and chronic low back pain: a systematic review and meta-analyses. Pain. 2022 Apr 1;163(4):610-636.
61. Tucker HR, Scaff K, McCloud T, Carlomagno K, Daly K, Garcia A, Cook CE. Harms and benefits of opioids for management of non-surgical acute and chronic low back pain: a systematic review. Br J Sports Med. 2020 Jun;54(11):664.
62. https://www.lakemedelsverket.se/sv/behandling-och-forskrivning/behandlingsrekommendationer/sok-behandlingsrekommendationer/lakemedel-vid-langvarig-smarta-hos-barn-och-vuxna--behandlingsrekommendation .
63. Giménez-Campos MS, Pimenta-Fermisson-Ramos P, Díaz-Cambronero JI, Carbonell-Sanchís R, López-Briz E, Ruíz-García V. A systematic review and meta-analysis of the effectiveness and adverse events of gabapentin and pregabalin for sciatica pain. Aten Primaria. 2022 Jan;54(1):102144.
64. Enke O, New HA, New CH, Mathieson S, McLachlan AJ, Latimer J, Maher CG, Lin CC. Anticonvulsants in the treatment of low back pain and lumbar radicular pain: a systematic review and meta-analysis. CMAJ. 2018 Jul 3;190(26):E786-E793.
65. Shanthanna H, Gilron I, Rajarathinam M, AlAmri R, Kamath S, Thabane L, Devereaux PJ, Bhandari M. Benefits and safety of gabapentinoids in chronic low back pain: A systematic review and meta-analysis of randomized controlled trials. PLoS Med. 2017 Aug 15;14(8):e1002369.
66. Ferreira GE, McLachlan AJ, Lin CC, Zadro JR, Abdel-Shaheed C, O'Keeffe M, Maher CG. Efficacy and safety of antidepressants for the treatment of back pain and osteoarthritis: systematic review and meta-analysis. BMJ. 2021 Jan 20;372:m4825.
67. Wielage R, Bansal M, Wilson K, Klein R, Happich M. Cost-effectiveness of duloxetine in chronic low back pain: a Quebec societal perspective. Spine (Phila Pa 1976). 2013 May 15;38(11):936-46.
68. Wielage RC, Bansal M, Andrews JS, Wohlreich MM, Klein RW, Happich M. The cost-effectiveness of duloxetine in chronic low back pain: a US private payer perspective. Value Health. 2013 Mar-Apr;16(2):334-44.
69. Cashin AG, Folly T, Bagg MK, Wewege MA, Jones MD, Ferraro MC, Leake HB, Rizzo RRN, Schabrun SM, Gustin SM, Day R, Williams CM, McAuley JH. Efficacy, acceptability, and safety of muscle relaxants for adults with non-specific low back pain: systematic review and meta-analysis. BMJ. 2021 Jul 7;374:n1446.

Systematic and critical review of reference literature:

| **Recommendations for investigations and assessment** | **Reference** | **Search strategy** | **Selection criteria** | **Assessors** | **AMSTER criteria** | | | | | | | | | | |
| --- | --- | --- | --- | --- | --- | --- | --- | --- | --- | --- | --- | --- | --- | --- | --- |
|  |  |  |  |  | **1** | **2** | **3** | **4** | **5** | **6** | **7** | **8** | **9** | **10** | **11** |
| 1 | Coeckelberghs etal. 2021 | Cochrane library, Pubmed, 17-11-2021  “Back pain and care pathways and international”. Limits: Systematic review | - Comparisons of back pain care pathways internationally  - Prioritise most recent Cochrane/SBU review over other reviews | AL, MK | 1 | 2 | 1 | 1 | 3 | 1 | 1 | 1 | 1 | 4 | 1 |
| 1 | Maselli etal. 2022 | Cochrane library, SBU, Pubmed, 07-04-2022  “Back pain and red flags”. Limits: Systematic review | - Back pain due to all serious conditions  - Prioritise most recent Cochrane/SBU review over other reviews | AA, GG | 1 | 1 | 1 | 1 | 1 | 1 | 1 | 1 | 1 | 3 | 1 |
| 1 | Fernando etal. 2022 | Cochrane library, SBU, Pubmed, 07-04-2022  “Back pain and abdominal aortic aneurysm”.  Limits: Systematic review | - Back pain due to Abdominal aortic aneurysm  - Prioritise most recent Cochrane/SBU review over other reviews | AA, ÅN | 1 | 1 | 1 | 1 | 3 | 1 | 1 | 1 | 1 | 3 | 1 |
| 1 | Dionne etal. 2019 | Cochrane library, SBU, Pubmed, 07-10-2022  “Back pain and cauda equina syndrome”.  Limits: Systematic review | - Back pain due to cauda equina syndrome  - Prioritise most recent Cochrane/SBU review over other reviews | AA, PE | 1 | 1 | 1 | 1 | 3 | 1 | 1 | 1 | 1 | 3 | 1 |
| 1 | Henschke etal. 2013 | Cochrane library, SBU, Pubmed, 07-10-2022  “Back pain and malignancy”  Limits: Systematic review | - Back pain due to malignancy  - Prioritise most recent Cochrane/SBU review over other reviews | AA, PF | 1 | 1 | 1 | 1 | 1 | 1 | 1 | 1 | 1 | 3 | 1 |
| 1 | Yusuf etal. 2019 | Cochrane library, SBU, Pubmed, 07-10-2022  “Back pain and infection”  Limits: Systematic review | - Back pain due to spinal infection  - Prioritise most recent Cochrane/SBU review over other reviews | AA, TT | 1 | 1 | 1 | 1 | 1 | 1 | 3 | 3 | 3 | 3 | 1 |
| 1 | Williams etal. 2013 | Cochrane library, Pubmed, 07-10-2022  “Back pain and vertebral fracture”  Limits: Systematic review | - Back pain due to vertebral fracture  - Prioritise most recent Cochrane/SBU review over other reviews | AA, SK | 1 | 1 | 1 | 1 | 1 | 1 | 1 | 1 | 1 | 3 | 1 |
| 1 | Sepriano etal. 2017 | Cochrane library, SBU, Pubmed, 07-10-2022  “ASAS criteria and spondylarthitis”  Limits: Systematic review | - Back pain due to spondyloarthritis  - Prioritise most recent Cochrane/SBU review over other reviews | AA, TO | 1 | 1 | 1 | 1 | 1 | 1 | 1 | 1 | 3 | 3 | 1 |
| 2 | Lim etal. 2019 | Cochrane library, SBU, Pubmed, 07-10-2022  “Back pain and patient perspective”  Limits: Systematic review | - Patient perspective of care for back pain  - Prioritise most recent Cochrane/SBU review over other reviews | AL, SL | 1 | 1 | 1 | 1 | 1 | 1 | 1 | 2 | 4 | 4 | 1 |
| 2 | Brinjikji etal. 2015 | Cochrane library, SBU, Pubmed, 07-10-2022  ”Asymptomatic and spine and imaging and features”  Limits: Systematic review | - Prevalence of asymptomatic spine features  - Prioritise most recent Cochrane/SBU review over other reviews | AA, ME | 1 | 1 | 1 | 1 | 3 | 1 | 2 | 2 | 4 | 4 | 1 |
| 2 | Raastad etal. 2015 | Cochrane library, SBU, Pubmed, 07-10-2022  ”Back pain and imaging and features”  Limits: Systematic review | - Association between back pain spine features  - Prioritise most recent Cochrane/SBU review over other reviews | AA, MF | 1 | 1 | 1 | 1 | 3 | 1 | 1 | 1 | 1 | 3 | 1 |
| 2 | Chou etal. 2009 | Cochrane library, SBU, Pubmed, 07-10-2022  ”Back pain and imaging strategy”  Limits: Systematic review | - Comparison of patient back pain outcomes with or without use of lumbar imaging  - Prioritise most recent Cochrane/SBU review over other reviews | AA, PF | 1 | 1 | 1 | 1 | 3 | 1 | 1 | 1 | 1 | 3 | 1 |
| 2 | Jenkins etal. 2018 | Cochrane library, SBU, Pubmed, 07-10-2022  ”Back pain and imaging and guideline consistency”  Limits: Systematic review | - Appropriateness of lumbar imaging for back pain  - Prioritise most recent Cochrane/SBU review over other reviews | AA, PE | 1 | 1 | 1 | 1 | 3 | 1 | 1 | 1 | 1 | 3 | 1 |
| 2 | Lemmers etal. 2019 | Cochrane library, SBU, Pubmed, 07-10-2022  ”Back pain and imaging and costs”  Limits: Systematic review | - Comparison of back pain cost outcomes with or without use of lumbar imaging  - Prioritise most recent Cochrane/SBU review over other reviews | AA, GG | 1 | 1 | 1 | 1 | 1 | 1 | 1 | 1 | 1 | 3 | 1 |
| 3 | SBU_UT202215 2022 | Cochrane library, SBU, Pubmed, 07-10-2022  ”Prediction and pain outcomes and primary care”  Limits: Systematic review | - Identification of patients in primary care at risk of worsening pain  - Prioritise most recent Cochrane/SBU review over other reviews | AA, TT | 1 | 1 | 1 | 1 | 1 | 1 | 1 | 1 | 1 | 3 | 1 |
| 3 | Karran etal 2017 | Cochrane library, SBU, Pubmed, 07-10-2022  ”Back pain and screening instruments and poor outcome”  Limits: Systematic review | - Prognostic accuracy of screening instruments for back pain related outcomes  - Prioritise most recent Cochrane/SBU review over other reviews | AA, TT | 1 | 1 | 1 | 1 | 1 | 1 | 1 | 1 | 1 | 3 | 1 |
| 3 | Ogbeivor & Elsabbagh 2021 | Cochrane library, SBU, Pubmed, 07-10-2022  ”Back pain and prognostic screening and targeted treatment”  Limits: Systematic review | - Effectiveness of targeted treatments based on prognostic for back pain related outcomes  - Prioritise most recent Cochrane/SBU review over other reviews | AA, TT | 1 | 1 | 1 | 1 | 1 | 1 | 1 | 1 | 1 | 3 | 1 |
| **Recommendation for first-line treatments** | **Reference** | **Search strategy** | **Selection criteria** | **Assessor** | **AMSTER criteria** | | | | | | | | | | |
|  |  |  |  |  | **1** | **2** | **3** | **4** | **5** | **6** | **7** | **8** | **9** | **10** | **11** |
| 1 | Jones etal. 2021 | Cochrane library, SBU, Pubmed, 07-10-2022  ”Back pain and prognostic screening and targeted treatment”  Limits: Systematic review | - Effectiveness of advice and education on back pain related outcomes  - Prioritise most recent Cochrane/SBU review over other reviews | SL, AL, AA | 1 | 1 | 1 | 1 | 1 | 1 | 1 | 1 | 1 | 3 | 1 |
| 1 | Andronius etal. 2017 | Cochrane library, SBU, Pubmed, 07-10-2022  ”Back pain and non-invasive interventions and cost-effectiveness ”  Limits: Systematic review | - Cost-effectiveness of non-invasive intervention for back pain.  - Prioritise most recent Cochrane/SBU review over other reviews | SL, AL, AA | 1 | 1 | 1 | 1 | 1 | 1 | 1 | 1 | 1 | 3 | 1 |
| 2 | Ho etal. 2022 | Cochrane library, SBU, Pubmed, 07-10-2022  ”Back pain and psychological interventions back pain and prevention and future impact”  Limits: Systematic review | - Effectiveness of psychological interventions for back pain.  - Prioritise most recent Cochrane/SBU review over other reviews | SL, MK, AA | 1 | 1 | 1 | 1 | 3 | 1 | 1 | 1 | 1 | 3 | 1 |
| 2 | de Campos etal. 2021 | Cochrane library, SBU, Pubmed, 07-10-2022  ”Back pain and preventions strategies and future impact”  Limits: Systematic review | - Effectiveness of prevention strategies for future back pain.  - Prioritise most recent Cochrane/SBU review over other reviews | SL, MK, AA | 1 | 1 | 1 | 1 | 3 | 1 | 1 | 1 | 1 | 3 | 1 |
| 2 | Rathnayake etal. 2021 | Cochrane library, SBU, Pubmed, 07-10-2022  ”Back pain and preventions strategies and future impact”  Limits: Systematic review | - Effectiveness of self-management combined with exercise for back pain.  - Prioritise most recent Cochrane/SBU review over other reviews | SL, MK, AA | 1 | 1 | 1 | 1 | 3 | 1 | 1 | 1 | 1 | 3 | 1 |
| 3 | Hayden etal. 2021, Cochrane Database Syst Rev | Cochrane library, SBU, Pubmed, 07-10-2022  ”Chronic Back pain and exercise therapy and effectiveness”  Limits: Systematic review | - Effectiveness of exercise therapy for chronic back pain.  - Prioritise most recent Cochrane/SBU review over other reviews | SL, MK, AA | 1 | 1 | 1 | 1 | 1 | 1 | 1 | 1 | 1 | 3 | 1 |
| 3 | Hayden etal. 2021, J Physiother | Cochrane library, SBU, Pubmed, 07-10-2022  ”Chronic Back pain and exercise therapy and effectiveness”  Limits: Systematic review | - Effectiveness of exercise therapy for chronic back pain.  - Prioritise most recent Cochrane/SBU review over other reviews | AA, TT | 1 | 1 | 1 | 1 | 1 | 1 | 1 | 1 | 1 | 3 | 1 |
| 3 | Karlsson etal. 2020 | Cochrane library, SBU, Pubmed, 07-10-2022  ”Acute Back pain and exercise therapy and effectiveness”  Limits: Systematic review | - Effectiveness of exercise therapy for acute back pain.  - Prioritise most recent Cochrane/SBU review over other reviews | SL, TT, AA | 1 | 1 | 1 | 1 | 1 | 1 | 1 | 1 | 1 | 3 | 1 |
| 3 | Miyamoto etal. 2019 | Cochrane library, SBU, Pubmed, 07-10-2022  ”Back pain and exercise therapy and cost-effectiveness”  Limits: Systematic review | - Cost-effectiveness of exercise therapy for back pain.  - Prioritise most recent Cochrane/SBU review over other reviews | SL, AA | 1 | 1 | 1 | 1 | 3 | 1 | 1 | 1 | 1 | 3 | 1 |
| **Recommendations for non-pharmacological adjunct treatments** | **Reference** | **Search strategy** | **Selection criteria** | **Assessor** | **AMSTER criteria** | | | | | | | | | | |
|  |  |  |  |  | **1** | **2** | **3** | **4** | **5** | **6** | **7** | **8** | **9** | **10** | **11** |
| 1 | Rubenstein etal. 2019 | Cochrane library, SBU, Pubmed, 07-10-2022  ”Chronic back pain and spinal manipulative therapy and effectiveness”  Limits: Systematic review | - Effectiveness of manual therapy for chronic back pain.  - Prioritise most recent Cochrane/SBU review over other reviews | AL, ME, AA | 1 | 1 | 1 | 1 | 1 | 1 | 1 | 1 | 1 | 3 | 1 |
| 1 | de Zoete etal. 2021 | Cochrane library, SBU, Pubmed, 07-10-2022  ”Chronic back pain and spinal manipulative therapy and effectiveness”  Limits: Systematic review | - Effectiveness of manual therapy for chronic back pain.  - Prioritise most recent Cochrane/SBU review over other reviews | AL, ME, AA | 1 | 1 | 1 | 1 | 1 | 1 | 1 | 1 | 1 | 3 | 1 |
| 1 | Rubenstein etal. 2012 | Cochrane library, SBU, Pubmed, 07-10-2022  ”Acute back pain and spinal manipulative therapy and effectiveness”  Limits: Systematic review | - Effectiveness of spinal manipulative therapy for acute back pain.  - Prioritise most recent Cochrane/SBU review over other reviews | AL, ME, AA | 1 | 1 | 1 | 1 | 1 | 1 | 1 | 1 | 1 | 3 | 1 |
| 1 | Paige etal 2017 | Cochrane library, SBU, Pubmed, 07-10-2022  ”Acute back pain AND spinal manipulative therapy AND effectiveness”  Limits: Systematic review | - Effectiveness of spinal manipulative therapy for acute back pain.  - Prioritise most recent Cochrane/SBU review over other reviews | AL, ME, AA | 1 | 1 | 1 | 1 | 1 | 1 | 1 | 1 | 1 | 3 | 1 |
| 2 | Su etal. 2021 | Cochrane library, SBU, Pubmed, 07-10-2022  ”(Back pain OR spinal pain) AND acupuncture AND (effectiveness OR efficacy)”  Limits: Systematic review | - Effectiveness of acupuncture for back pain.  - Prioritise most recent Cochrane/SBU review over other reviews | TT, MF, AA | 1 | 1 | 1 | 1 | 3 | 1 | 1 | 1 | 1 | 3 | 1 |
| 2 | Xiang etal 2020 | Cochrane library, SBU, Pubmed, 07-10-2022  ”(Back pain OR spinal pain) AND acupuncture AND (effectiveness OR efficacy)”  Limits: Systematic review | - Effectiveness/efficacy of acupuncture for back pain.  - Prioritise most recent Cochrane/SBU review over other reviews | TT, MK, AA | 1 | 1 | 1 | 1 | 3 | 1 | 1 | 1 | 1 | 3 | 1 |
| 2 | Baroncini etal 2022 | Cochrane library, SBU, Pubmed, 07-10-2022  ”(Back pain OR spinal pain) AND acupuncture AND (effectiveness OR efficacy)”  Limits: Systematic review | - Effectiveness/efficacy of acupuncture for back pain.  - Prioritise most recent Cochrane/SBU review over other reviews | TT, MK, AA | 1 | 1 | 1 | 1 | 3 | 1 | 1 | 1 | 1 | 3 | 1 |
| 2 | Asano etal 2022 | Cochrane library, SBU, Pubmed, 07-10-2022  ”(Back pain OR spinal pain) AND acupuncture AND (effectiveness OR efficacy)”  Limits: Systematic review | - Effectiveness/efficacy of acupuncture for back pain.  - Prioritise most recent Cochrane/SBU review over other reviews | TT, MK, AA | 1 | 3 | 3 | 1 | 3 | 1 | 1 | 1 | 1 | 3 | 1 |
| 2 | Huang etal 2021 | Cochrane library, SBU, Pubmed, 07-10-2022  ”(Back pain OR spinal pain) AND acupuncture AND (effectiveness OR efficacy)”  Limits: Systematic review | - Effectiveness/efficacy of acupuncture for back pain.  - Prioritise most recent Cochrane/SBU review over other reviews | TT, MK, AA | 1 | 1 | 1 | 1 | 1 | 1 | 1 | 1 | 1 | 1 | 1 |
| 2 | Li etal 2020 | Cochrane library, SBU, Pubmed, 07-10-2022  ”(Back pain OR spinal pain) AND acupuncture AND (effectiveness OR efficacy)”  Limits: Systematic review | - Effectiveness/efficacy of acupuncture for back pain.  - Prioritise most recent Cochrane/SBU review over other reviews | TT, MF, AA | 1 | 1 | 1 | 1 | 3 | 1 | 1 | 1 | 1 | 3 | 1 |
| 2 | Mu etal 2020 | Cochrane library, SBU, Pubmed, 07-10-2022  ”(Back pain OR spinal pain) AND acupuncture AND (effectiveness OR efficacy)”  Limits: Systematic review | - Effectiveness/efficacy of acupuncture for back pain.  - Prioritise most recent Cochrane/SBU review over other reviews | TT, MK, AA | 1 | 1 | 1 | 1 | 1 | 1 | 1 | 1 | 1 | 1 | 1 |
| 3 | Binny etal 2019 | Cochrane library, SBU, Pubmed, 07-10-2022  ”(Back pain OR spinal pain) AND TENS AND (effectiveness OR efficacy)”  Limits: Systematic review | - Effectiveness/efficacy of TENS for acute back pain.  - Prioritise most recent Cochrane/SBU review over other reviews | TT, MK, AA | 1 | 1 | 1 | 1 | 3 | 1 | 1 | 1 | 1 | 3 | 1 |
| 3 | Wu etal 2018 | Cochrane library, SBU, Pubmed, 07-10-2022  ”(Back pain OR spinal pain) AND TENS AND (effectiveness OR efficacy)”  Limits: Systematic review | - Effectiveness/efficacy of TENS for chronic back pain.  - Prioritise most recent Cochrane/SBU review over other reviews | TT, MK, AA | 1 | 1 | 1 | 1 | 3 | 1 | 1 | 1 | 1 | 1 | 1 |
| 4 | Wegner etal 2013 | Cochrane library, SBU, Pubmed, 07-10-2022  ”(Back pain OR spinal pain) AND Traction AND (effectiveness OR efficacy)”  Limits: Systematic review | - Effectiveness/efficacy of traction for back pain.  - Prioritise most recent Cochrane/SBU review over other reviews | AA, TT | 1 | 1 | 1 | 1 | 1 | 1 | 1 | 1 | 1 | 1 | 1 |
| 4 | Vanti etal 2021 | Cochrane library, SBU, Pubmed, 07-10-2022  ”Lumbar radiculopathy AND Traction AND (effectiveness OR efficacy)”  Limits: Systematic review | - Effectiveness/efficacy of traction for lumbar radiculopathy.  - Prioritise most recent Cochrane/SBU review over other reviews | AA, TT | 1 | 1 | 1 | 1 | 1 | 1 | 1 | 1 | 1 | 1 | 1 |
| 4 | Cheng etal 2020 | Cochrane library, SBU, Pubmed, 07-10-2022  ”Disc herniation AND Traction AND (effectiveness OR efficacy)”  Limits: Systematic review | - Effectiveness/efficacy of traction for disc herniation.  - Prioritise most recent Cochrane/SBU review over other reviews | TT, MK, AA | 1 | 1 | 1 | 1 | 3 | 1 | 1 | 1 | 1 | 1 | 1 |
| 5 | Gignoux etal 2022 | Cochrane library, SBU, Pubmed, 07-10-2022  ”(Back pain OR spinal pain) AND lumbar support AND (effectiveness OR efficacy)”  Limits: Systematic review | - Effectiveness/efficacy of lumbar supports for back pain.  - Prioritise most recent Cochrane/SBU review over other reviews | TT, MK, AA | 1 | 1 | 1 | 1 | 1 | 1 | 1 | 1 | 1 | 3 | 1 |
| 5 | Kong etal 2020 | Cochrane library, SBU, Pubmed, 07-10-2022  ”(Back pain OR spinal pain) AND shoe insoles AND (effectiveness OR efficacy)”  Limits: Systematic review | - Effectiveness/efficacy of shoe insoles for back pain.  - Prioritise most recent Cochrane/SBU review over other reviews | TT, MK, AA | 1 | 1 | 1 | 1 | 3 | 1 | 1 | 1 | 1 | 2 | 1 |
| 5 | Ebadi etal 2020 | Cochrane library, SBU, Pubmed, 07-10-2022  ”(Back pain OR spinal pain) AND ultrasound AND (effectiveness OR efficacy)”  Limits: Systematic review | - Effectiveness/efficacy of ultrasound for back pain.  - Prioritise most recent Cochrane/SBU review over other reviews | TT, MK, AA | 1 | 1 | 1 | 1 | 1 | 1 | 1 | 1 | 1 | 1 | 1 |
| **Recommendations for pharmacological adjunct treatment** | **Reference** | **Search strategy** | **Selection criteria** |  | **AMSTER** | | | | | | | | | | |
|  |  |  |  |  | **1** | **2** | **3** | **4** | **5** | **6** | **7** | **8** | **9** | **10** | **11** |
| 1 | Machado etal 2017 | Cochrane library, SBU, Pubmed, 07-10-2022  ”(Back pain OR spinal pain) AND nonsteroidal anti-inflammatory drugs AND (effectiveness OR efficacy)”  Limits: Systematic review | - Effectiveness/efficacy of nonsteroidal anti-inflammatory drugs for back/spinal pain.  - Prioritise most recent Cochrane/SBU review over other reviews | GG, ÅN, AA | 1 | 1 | 1 | 1 | 3 | 1 | 1 | 1 | 1 | 1 | 1 |
| 1 | van der Gaag etal 2020 | Cochrane library, SBU, Pubmed, 07-10-2022  ”(Back pain OR spinal pain) AND acute AND nonsteroidal anti-inflammatory drugs AND (effectiveness OR efficacy)”  Limits: Systematic review | - Effectiveness/efficacy of nonsteroidal anti-inflammatory drugs for acute back/spinal pain.  - Prioritise most recent Cochrane/SBU review over other reviews | GG, ÅN, AA | 1 | 1 | 1 | 1 | 1 | 1 | 1 | 1 | 1 | 1 | 1 |
| 1 | Enthoven etal 2026 | Cochrane library, SBU, Pubmed, 07-10-2022  ”(Back pain OR spinal pain) AND chronic AND nonsteroidal anti-inflammatory drugs AND (effectiveness OR efficacy)”  Limits: Systematic review | - Effectiveness/efficacy of nonsteroidal anti-inflammatory drugs for chronic back/spinal pain.  - Prioritise most recent Cochrane/SBU review over other reviews | GG, ÅN, AA | 1 | 1 | 1 | 1 | 1 | 1 | 1 | 1 | 1 | 1 | 1 |
| 2 | Saragiotto etal 2016 | Cochrane library, SBU, Pubmed, 07-10-2022  ”(Back pain OR spinal pain) AND paracetamol AND (effectiveness OR efficacy)”  Limits: Systematic review | - Effectiveness/efficacy of paracetamol for back/spinal pain.  - Prioritise most recent Cochrane/SBU review over other reviews | GG, ÅN, AA | 1 | 1 | 1 | 1 | 1 | 1 | 1 | 1 | 1 | 1 | 1 |
| 3 | Sanger etal 2019 | Cochrane library, SBU, Pubmed, 07-10-2022  ”(Back pain OR spinal pain) AND opiods AND (effectiveness OR efficacy)”  Limits: Systematic review | - Effectiveness/efficacy of opiods for acute back/spinal pain.  - Prioritise most recent Cochrane/SBU review over other reviews | GG, PE, AA | 1 | 1 | 1 | 1 | 3 | 1 | 1 | 1 | 1 | 3 | 1 |
| 3 | Nury etal 2022 | Cochrane library, SBU, Pubmed, 07-10-2022  ”(Back pain OR spinal pain) AND opiods AND (effectiveness OR efficacy)”  Limits: Systematic review | - Effectiveness/efficacy of opiods for chronic back/spinal pain.  - Prioritise most recent Cochrane/SBU review over other reviews | ÅN, PE, AA | 1 | 1 | 1 | 1 | 3 | 1 | 1 | 1 | 1 | 1 | 1 |
| 3 | Tucker etal 2020 | Cochrane library, SBU, Pubmed, 07-10-2022  ”(Back pain OR spinal pain) AND opiods AND (effectiveness OR efficacy)”  Limits: Systematic review | - Effectiveness/efficacy of opiods for back/spinal pain.  - Prioritise most recent Cochrane/SBU review over other reviews | PE, TT, AA | 1 | 1 | 1 | 1 | 3 | 1 | 1 | 1 | 1 | 3 | 1 |
| 4 | Giménez-Campos etal 2022 | Cochrane library, SBU, Pubmed, 07-10-2022  ”(Lumbar radicular pain OR sciatica) AND (Gabapentinoids OR antiepileptics OR anticonvulsants) AND (effectiveness OR efficacy)”  Limits: Systematic review | - Effectiveness/efficacy of gabapentinoids for sciatica/lumbar radicular pain.  - Prioritise most recent Cochrane/SBU review over other reviews | TT, PE, AA | 1 | 1 | 1 | 1 | 1 | 1 | 1 | 1 | 1 | 2 | 1 |
| 4 | Enke etal 2018 | Cochrane library, SBU, Pubmed, 07-10-2022  ”(Lumbar radicular pain OR sciatica) AND (Gabapentinoids OR antiepileptics OR anticonvulsants) AND (effectiveness OR efficacy)”  Limits: Systematic review | - Effectiveness/efficacy of gabapentinoids for sciatica/lumbar radicular pain.  - Prioritise most recent Cochrane/SBU review over other reviews | PE, ÅN, AA | 1 | 1 | 1 | 1 | 3 | 1 | 1 | 1 | 1 | 2 | 1 |
| 4 | Shanthanna etal 2017 | Cochrane library, SBU, Pubmed, 07-10-2022  ”(back pain OR spinal pain) AND (Gabapentinoids OR antiepileptics OR anticonvulsants) AND (effectiveness OR efficacy)”  Limits: Systematic review | - Effectiveness/efficacy of gabapentinoids for chronic back/spinal pain.  - Prioritise most recent Cochrane/SBU review over other reviews | AA  PE | 1 | 1 | 1 | 1 | 2 | 1 | 1 | 1 | 1 | 1 | 1 |
| 5 | Ferreira etal 2021 | Cochrane library, SBU, Pubmed, 07-10-2022  “(back pain OR spinal pain) AND (antidepressants) AND (effectiveness OR efficacy)”. Limits: Systematic review | - Effectiveness/efficacy of antidepressants for back/spinal pain.  - Prioritise most recent Cochrane/SBU review over other reviews | AA  PE | 1 | 1 | 1 | 1 | 1 | 1 | 1 | 1 | 1 | 1 | 1 |
| 6 | Cashin etal 2021 | Cochrane library, SBU, Pubmed, 07-10-2022  “(back pain OR spinal pain) AND (muscle relaxants) AND (effectiveness OR efficacy)”  Limits: Systematic review | - Effectiveness/efficacy of antidepressants for back/spinal pain.  - Prioritise most recent Cochrane/SBU review over other reviews | MK  PE | 1 | 1 | 1 | 1 | 1 | 1 | 1 | 1 | 1 | 1 | 1 |
