# Supplementary material for: Development of a Person-Centred Coordinated Care Pathway in Swedish Healthcare for Low Back Pain
Source: Int J Integr Care. 2025 May 9;25(2):8. doi: 10.5334/ijic.8940 (PMC12063581; doi:10.5334/ijic.8940)
Supplement: Appendices. — Appendix A–K. [file ijic-25-2-8940-s1.zip › ijic-8940_abbott-s4.pdf]

Appendix D. Advice and referral support

Immediately<br>Assessment should take place immediately. Acute referral to emergency services.	• Affected general condition associated with back pain, often accompanied by fever and chills, can be symptoms of serious infection.<br>• Non-mechanical back pain and pain from the abdomen or chest: Back pain that is not affected by movement, with or without concomitant chest or abdominal symptoms, can be symptoms of, for example, kidney stones, gallstones, myocardial infarction or rupture of the aorta. Other concomitant symptoms may include shortness of breath, cold sweats and anxiety.<br>• Sudden back pain with shortness of breath: may be due to a pulmonary embolism or a pneumothorax. Pneumothorax can occur either spontaneously or from trauma to the chest. Pneumothorax can also provide a feeling of stomach pain. Factors that increase the risk of blood clots are, for example, immobility, recent surgery, tumor disease, pregnancy and contraceptive pills.<br>• Difficulty controlling bladder or bowel, or other neurological symptoms: may be symptoms of a spinal cord disorder (cauda equina syndrome). It can also cause reduced sensation around the rectum and genitals and reduced sensation in the legs and feet.<br>• Severe back pain after trauma.
Urgently<br>The assessment must take place within one to 10 hours. Referral to emergency services.	• Back pain and fever can be caused by inflammation of the lungs, urinary tract, ovaries or prostate. It can also be symptoms of an infection in the vertebra or disc.
Within 24 hours<br>An assessment will be made within the next 24 hours. Referral to primary care or emergency services.	• Severe pain in the back or flank despite self-care: sometimes with restriction of movement or with intense pain that radiates to one leg and where self-care does not have sufficient effect should be assessed.
In the near future<br>Assessment should take place within the next week, or in some cases, in the next few weeks. Referral to primary health care.	• Back pain despite self-care: can be caused by prolonged sitting or unsatisfactory working posture. One can also get pain from over-exertion. Another cause may be vertebral compression even without trauma in osteoporosis or tumor cases. If the patient has tried self-care for two to three weeks without sufficient effect, the patient should be assessed. Constant or increasing night-time back pain, previous/ongoing cancer and involuntary weight loss may be due to a spinal tumor and should be evaluated. At 4 out of 5 of the following criteria: 1) Younger than 40 years, 2) Insidious onset of back pain, 3) Improvement of symptoms with movement, 4) No improvement of symptoms with rest, 5) Night pain, may be signs on ankylosing spondylitis and the patient should receive a physical assessment.
Wait<br>The applicant can wait for another healthcare contact. Often the patient is helped by advice on self-care.	• Back pain that is considered to be temporary. If the patient can manage daily tasks, they can wait with the help from healthcare and instead continue self-care for two weeks.

References

https://hanvisning.sll.se/documents/5cc61cb10b6275d3b0279f2d
https://www.1177.se/om-1177-vardguiden/om-1177-vardguiden/radgivningsstodet-webb--ett-stod-i-din-verksamhet/
