# Supplementary material for: Development of a Person-Centred Coordinated Care Pathway in Swedish Healthcare for Low Back Pain
Source: Int J Integr Care. 2025 May 9;25(2):8. doi: 10.5334/ijic.8940 (PMC12063581; doi:10.5334/ijic.8940)
Supplement: Appendices. — Appendix A–K. [file ijic-25-2-8940-s1.zip › ijic-8940_abbott-s5.pdf]

Appendix E – Screening for red flags (questions to patient with low back problems)

Screening of medical permission	Questions for patients	Response	
Kidney stones, gallstones, myocardial infarction, aortic aneurysm	Do you have severe back pain that is not affected by movement with simultaneous symptoms from chest or abdomen?	YES	NO
Pulmonary embolism	Do you have sudden back pain with shortness of breath?	YES	NO
Cauda equina/spinal cord injury	Do you have back pain along with difficulty emptying/controlling your bladder and bowels?	YES	NO
Cauda equina/spinal cord injury	Do you have back pain along with altered sensation in genital area or around the rectum?	YES	NO
Cauda equina/spinal cord injury	Do you have back pain along with loss of strength and/or feeling in the legs?	YES	NO
Fracture	Have you had severe back pain after a trauma or fall?	YES	NO
Infection	Do you have a general feeling of malaise in connection with back pain?	YES	NO
Infection	Do you have back pain along with a fever that comes and goes?	YES	NO
Cancer	Have you previously been diagnosed with or treated for cancer?	YES	NO
Cancer	Have you lost weight involuntarily?	YES	NO
AS*	Are you younger than 40?	YES	NO
AS*	Has the back pain had a insidious debut?	YES	NO
AS*	Does moving around improve back pain?	YES	NO
AS*	Do you get no improvement in your back pain when you rest?	YES	NO
AS*	Do you have back pain during the night?	YES	NO
* Ankylosing spondylitis (AS) is suspected if 4 out of 5 criteria are met.			

References

- Maselli F, Palladino M, Barbari V, Storari L, Rossettini G, Testa M. The diagnostic value of Red Flags in thoracolumbar pain: a systematic review. Disabil Rehabil. 2022 Apr;44(8):1190-1206.
- Fernando SM, Tran A, Cheng W, Rochwerg B, Strauss SA, Mutter E, McIsaac DI, Kyeremanteng K, Kubelik D, Jetty P, Nagpal SK, Thiruganasambandamoorthy V, Roberts DJ, Perry JJ. Accuracy of presenting symptoms, physical examination, and imaging for diagnosis of ruptured abdominal aortic aneurysm: Systematic review and meta-analysis. Acad Emerg Med. 2022 Apr;29(4):486-496.
- Dionne N, Adefolarin A, Kunzelman D, Trehan N, Finucane L, Levesque L, Walton DM, Sadi J. What is the diagnostic accuracy of red flags related to cauda equina syndrome (CES), when compared to Magnetic Resonance Imaging (MRI)? A systematic review. Musculoskelet Sci Pract. 2019 Jul;42:125- 133.
- Henschke N, Maher CG, Ostelo RW, de Vet HC, Macaskill P, Irwig L. Red flags to screen for malignancy in patients with low back pain. Cochrane Database Syst Rev 2013; 2: CD008686.
- Yusuf M, Finucane L, Selfe J. Red flags for the early detection of spinal infection in back pain patients. BMC Musculoskelet Disord. 2019 Dec 13;20(1):606.
- Williams CM, Henschke N, Maher CG, van Tulder MW, Koes BW, Macaskill P, Irwig L. Red flags to screen for vertebral fracture in patients presenting with low-back pain. Cochrane Database Syst Rev 2013; 1: CD008643.
- Sepriano A, Rubio R, Ramiro S, Landewé R, van der Heijde D. Performance of the ASAS classification criteria for axial and peripheral spondyloarthritis: a systematic literature review and meta-analysis. Ann Rheum Dis. 2017 May;76(5):886-890
