# Supplementary material for: Development of a Person-Centred Coordinated Care Pathway in Swedish Healthcare for Low Back Pain
Source: Int J Integr Care. 2025 May 9;25(2):8. doi: 10.5334/ijic.8940 (PMC12063581; doi:10.5334/ijic.8940)
Supplement: Appendices. — Appendix A–K. [file ijic-25-2-8940-s1.zip › ijic-8940_abbott-s6.docx]

Appendix F – Investigation of yellow and blue flags (psychosocial and work-related factors)

Use for example, [ÖMPSQ-short](https://www.oru.se/globalassets/oru-en/research/environments/hs/jps/champ/ompsq-short-version-eng.pdf) or [Start Back Screening Tool (SBT)](https://startback.hfac.keele.ac.uk/wp-content/uploads/2018/11/Keele_STarT_Back9_item.pdf)

- ÖMPSQ: A questionnaire about pain problems (short version): 10 questions (0–100 points) that address how the problems affect the patient regarding movement function, pain, psychological factors, coping skills, fear avoidance and work ability. It enables classification according to risk level. High score >50 points, is high risk of developing long-term disabilities and sick leave, 40–49 points is moderate risk and below 40 points is low risk. The questions can be used as a basis for a dialogue with the patient [Linton etal 2011].
- Start Back Screening Tool (SBT): A risk stratification tool comprising 9 questions (0–9 points) specifically used to classify patients according to their risk of long-term back pain into three different risk groups based on modifiable physical and psychosocial risk factors. SBT is a scientifically quality-assured tool where clear action strategies have been defined for each risk group and its use has proven to be a cost-effective strategy in primary care [Hill etal 2008]. The questions are divided into physical risk factors (referred leg pain, comorbid pain, difficulty walking and dressing) and a psychosocial subscale with psychosocial risk factors (fear avoidance, anxiety, catastrophizing, depression and overall impact of back pain). Low total score (0–3p) = Low risk, medium total score (≥ 4p) = Moderate risk, high total score on psychosocial subscale (≥ 4p) = High risk [Forsbrand etal 2017].
- Both forms aim to identify patients at risk for long-term disability based on the interaction between complaints and psychosocial factors [Karran etal 2017]. They cannot replace the history or the physical examination. They are aids aimed at early identification of patients who are at risk of developing long-term disability or sickness absence related to pain. The forms are a basis for discussion for caregivers and patients to see the whole and how the pain affects the patient in everyday life.
- At low risk, plan treatment based on recommended actions such as information, self-care for pain relief, physical exercise and return to a full activity level.
- In case of higher risk according to screening, investigate the following:
  - Work ability score: We assume that your work ability, when it was at its best, is valued at 10 points. What score would you then give your current work ability?”
  - Map possible stress factors and investigate predictors of return to work. For example, through Workplace Dialogue for Return to Work (ADA). ADA is a structured dialogue in three stages between the patient, the healthcare professional and the workplace. The aim is to find concrete suggestions that can support the patient to maintain work ability, and if the patient is on sick leave, to return to work. ADA, when added to structured physiotherapy, has been shown to improve work ability after one year for people with acute/subacute back pain in Swedish primary care [Sennehed etal 2018] and it has also been shown to be a cost-effective method [Saha etal 2019].
  - Investigate possible psychological factors such as depression, anxiety and psychiatric comorbidity.
  - Map unhealthy lifestyle behaviours according to the National Board of Health and Welfare's guidelines. [Nationella riktlinjer för prevention och behandling vid ohälsosamma levnadsvanor (socialstyrelsen.se)](https://www.socialstyrelsen.se/globalassets/sharepoint-dokument/artikelkatalog/nationella-riktlinjer/2018-6-24.pdf)
  - Investigate possible effects on sleep. For example, the [Insomnia Severity Index](https://www.healthquality.va.gov/guidelines/CD/insomnia/TrifectaInsomniaSeverityIndexFillable910162020.pdf)
  - Investigate possible social factors: abuse, bullying/threats, family stress.

References:

- Linton SJ, Nicholas M, MacDonald S. Development of a Short Form of the Örebro Musculoskeletal Pain Screening Questionnaire. Spine. 2011 36, 1891–1895.
- Hill JC, Dunn KM, Lewis M, Mullis R, Main CJ, Foster NE, Hay EM. A primary care back pain screening tool: identifying patient subgroups for intitial treatment. Arthrithis Rheum.2008;59(5):632-41.
- Forsbrand M, Grahn B, Hill JC, Petersson IF, Sennehed CP, Stigmar K. Comparison of the Swedish STarT Back Screening Tool and the Short Form of the Örebro Musculoskeletal Pain Screening Questionnaire in patients with acute or subacute back and neck pain. BMC Musculoskelet Disord. 2017 Feb 21;18(1):89.
- Karran EL, McAuley JH, Traeger AC, Hillier SL, Grabherr L, Russek LN, Moseley GL. Can screening instruments accurately determine poor outcome risk in adults with recent onset low back pain? A systematic review and meta-analysis. BMC Med. 2017 Jan 19;15(1):13.
- Sennehed CP, Holmberg S, Axén I, Stigmar K, Forsbrand M, Petersson IF, Grahn B. Early workplace dialogue in physiotherapy practice improved work ability at 1-year follow-up - WorkUp, a randomised controlled trial in primary care. Pain. 2018 Aug;159(8):1456-1464.
- Saha S, Grahn B, Gerdtham UG, Stigmar K, Holmberg S, Jarl J. Structured physiotherapy including a workplace intervention for patients with neck and/or back pain in primary care: an economic evaluation. Eur J Health Econ. 2019 Mar;20(2):317-327.
