# Supplementary material for: Development of a Person-Centred Coordinated Care Pathway in Swedish Healthcare for Low Back Pain
Source: Int J Integr Care. 2025 May 9;25(2):8. doi: 10.5334/ijic.8940 (PMC12063581; doi:10.5334/ijic.8940)
Supplement: Appendices. — Appendix A–K. [file ijic-25-2-8940-s1.zip › ijic-8940_abbott-s7.pdf]

Appendix G. Generic subjective and objective assessment proforma

<table>
<tr><td rowspan="2">Subjective assessment</td><td>Date:</td><td colspan="2">Patient number:</td><td>Name:</td><td>Other details:</td></tr>
<tr><td>Height:</td><td>Weight:</td><td>BMI:</td><td>Employment and activity grade (%):</td><td>Sick leave (length and grade %):</td></tr>
<tr><td>Current back pain</td><td colspan="5">Onset, course, duration, character, intensity, frequency (constant/intermittent), 24hr variation (am/em/night), easily/not easily provoked, better/worse of:</td></tr>
<tr><td>Current leg pain</td><td colspan="5">Onset, course, duration, character, intensity, frequency (constant/intermittent), 24hr variation (am/em/night), easily/not easily provoked, better/worse of:</td></tr>
<tr><td>Other pain locations</td><td colspan="5">Onset, course, duration, character, intensity, frequency (constant/intermittent), 24hr variation (am/em/night), easily/not easily provoked, better/worse of:</td></tr>
<tr><td>Functional impairment</td><td colspan="5">Neurological deficit motor/sensory (weakness, numbness), movement/stiffness, instability:</td></tr>
<tr><td>Activity Limitation</td><td colspan="5">Work, leisure (incl. walking):</td></tr>
<tr><td>Yellow/Blue flags</td><td colspan="5">Pain behaviour/management, expectations for recovery, anxiety/stress, depression, factors affecting work ability:</td></tr>
<tr><td>Red flags</td><td colspan="5">Trauma, previous/ongoing cancer, unexplained weight loss, malaise, impaired bladder and bowel function, anaesthesia in genital/rectal area, chest or abdominal pain, shortness of breath:</td></tr>
<tr><td>Previous investigations</td><td colspan="5">Healthcare provider, type (X-ray, CT, MRI, EMG, blood tests), date and findings:</td></tr>
<tr><td>Previous treatments</td><td colspan="5">Healthcare provider, type of treatment (rehabilitation, analgesics, previous surgery) and results:</td></tr>
<tr><td>Past and present illnesses/injuries</td><td colspan="5">Type, how long:</td></tr>
<tr><td rowspan="2">Medications</td><td colspan="3" rowspan="2">Type, dose:</td><td colspan="2">Medication allergies</td></tr>
<tr><td colspan="2">Type:</td></tr>
<tr><td>Lifestyle</td><td colspan="5">Physical activity, sleep, eating habits, tobacco, alcohol:</td></tr>
</table>

**Physical assessment**	**Type and finding**	**Level/side**
General status	E.g. Atrophies, posture, functional body movements, walking ability, psyche, blood pressure, auscultation heart/respiration, circ (cyanosis, oedema, skin slumps, peripheral pulsars), abdominal palp, temp, skin:	
Neurological	E.g. PNS - Myotomes/dermatomes/reflexes, ischias/femoral stretch bilaterally, foramen comp test, CNS - Babinski/clonus, balance:	
Lumbar spine status	E.g. Inspect in resting position (olisthesis sign, asymmetry), symptom during static/repeated spinal movements (flex, ext, lat flex, rot), ROM, segmental pain provocation test, dorsal/ventral musc activation/tonus and endurance:	
SI-joint status	E.g. SI-joint pain provocation test, active SLR:	
Hip joint status	E.g. Movement, pain provocation test (FABER, FADIR):	
Yellow flags	E.g. Pain behaviour during physical assessment:	
Blue flags	E.g. Assessment of work ability:	

**Other assessments**	**Type och findings**
Medical imaging	E.g. X-ray, CT, MRI:
Laboratory tests	E.g. blood tests, CRP etc:
Electrophysiological tests	

**Summary**		**ICD-10 diagnosis**
Segmental nociceptive pain		
Neuropathic pain		
Nociplastic pain		
Other		

**Intervention – Rehabilitation plan**		**KVÅ code**
What should the patient do?		
What should the health care giver do?		
Goals och time plan?		
