# Supplementary material for: Development of a Person-Centred Coordinated Care Pathway in Swedish Healthcare for Low Back Pain
Source: Int J Integr Care. 2025 May 9;25(2):8. doi: 10.5334/ijic.8940 (PMC12063581; doi:10.5334/ijic.8940)
Supplement: Appendices. — Appendix A–K. [file ijic-25-2-8940-s1.zip › ijic-8940_abbott-s8.docx]

Appendix H. Proposals for patient-reported evaluation instruments

Basic evaluation:

- At baseline and follow-up:
  - Numeric rating scale - NRS back pain in the last week 0–10 from none to maximum pain.
  - Numeric rating scale - NRS leg pain in the last week 0–10 from none to maximum pain.
- At follow-up: What is your attitude towards the results of your completed treatment?
  1. I am satisfied 2. I am doubtful 3. I am dissatisfied.

Extended evaluation:

- At baseline and follow-up:
  - [Oswestry Disability Index (ODI):](https://www.medscinet.com/swespine/uploads/swespine-oswestry.pdf) a validated functional measurement för evaluating low back pain. 10 questions giving a maximum of 100 points. High scores indicate poorer functioning. A change of 20–40 points indicate improved function.
  - [EuroQol (EQ-5D):](https://www.medscinet.com/swespine/uploads/swespine-eq5d.pdf) a standardised measure of health-related quality of life. The EQ-5D descriptive system comprises five dimensions: mobility, self-care, usual activities, pain and discomfort, and anxiety and depression. In addition, a vertical VAS where 0 is low and 100 is high quality of life.
  - [Work Ability Score:](https://fhvmetodik.se/wp-content/uploads/2013/08/Work_Ability_Index_Formular_1_1.pdf) Question 1 is validated to be used as an indicator.
    "We assume that your work ability, when it was at its best, is valued with 10 points. What score would you then give your current work ability?”
  - [Patient Specific Function Scale (PSFS)](https://www.google.com/url?sa=t&rct=j&q=&esrc=s&source=web&cd=&ved=2ahUKEwjX9pC3mo35AhUIx4sKHT8xBqYQFnoECBwQAQ&url=https%3A%2F%2Fedilprod.dll.se%2FGetPublicFile.ashx%3Fdocid%3D556421&usg=AOvVaw2LK1iRRD3W590cAI_iD_es) based on degree of difficulty on a scale from 0-10, where 0 corresponds to "Can't perform the activity" and 10 stands for "Can perform the activity unhindered or as before the injury/disease". The PSFS identifies both physical, psychological, and social activity limitations.
