# Supplementary material for: Development of a Person-Centred Coordinated Care Pathway in Swedish Healthcare for Low Back Pain
Source: Int J Integr Care. 2025 May 9;25(2):8. doi: 10.5334/ijic.8940 (PMC12063581; doi:10.5334/ijic.8940)
Supplement: Appendices. — Appendix A–K. [file ijic-25-2-8940-s1.zip › ijic-8940_abbott-s9.docx]

Appendix I. Recommended ICD-10-codes for P3C pathway

| **Diagnoses based on symptoms** | **ICD-10 codes** |
| --- | --- |
| Lumbago with sciatica | M54.4 |
| Low back pain | M54.5 |
| Sciatica | M54.3 |
| Dorsalgia, unspecified | M54.9 |
| Other chronic pain | R52.2 |
| **Diagnoses based on structural pathology after extended examination** | **ICD-10 codes** |
| Spondylolysis | M43.0 |
| Spondylolisthesis | M43.1 |
| Deforming dorsopathy, unspecified | M43.9 |
| Spondylosis, unspecified | M47.9 |
| Spinal stenosis | M48.0 |
| Other specified spondylopathies | M48.8 |
| Spondylopathy, unspecified | M48.9 |
| Lumbar and other intervertebral disc disorders with myelopathy | M51.0 |
| Lumbar and other intervertebral disc disorders with radiculopathy | M51.1 |
| Other specified intervertebral disc displacement | M51.2 |
| Other specified intervertebral disc degeneration | M51.3 |
| Schmorl nodes | M51.4 |
| Other specified intervertebral disc disorders | M51.8 |
| Intervertebral disc disorder, unspecified | M51.9 |
| Spinal instabilities | M53.2 |
| Sacrococcygeal disorders, not elsewhere classified | M53.3 |
| Other specified dorsopathies | M53.8 |
| Dorsopathy, unspecified | M53.9 |
