# Supplementary material for: Development of a Person-Centred Coordinated Care Pathway in Swedish Healthcare for Low Back Pain
Source: Int J Integr Care. 2025 May 9;25(2):8. doi: 10.5334/ijic.8940 (PMC12063581; doi:10.5334/ijic.8940)
Supplement: Appendices. — Appendix A–K. [file ijic-25-2-8940-s1.zip › ijic-8940_abbott-s9.pdf]

Appendix I. Recommended ICD-10-codes for P3C pathway

Diagnoses based on symptoms	ICD-10 codes
Lumbago with sciatica	M54.4
Low back pain	M54.5
Sciatica	M54.3
Dorsalgia, unspecified	M54.9
Other chronic pain	R52.2
**Diagnoses based on structural pathology after extended examination**	**ICD-10 codes**
Spondylolysis	M43.0
Spondylolisthesis	M43.1
Deforming dorsopathy, unspecified	M43.9
Spondylosis, unspecified	M47.9
Spinal stenosis	M48.0
Other specified spondylopathies	M48.8
Spondylopathy, unspecified	M48.9
Lumbar and other intervertebral disc disorders with myelopathy	M51.0
Lumbar and other intervertebral disc disorders with radiculopathy	M51.1
Other specified intervertebral disc displacement	M51.2
Other specified intervertebral disc degeneration	M51.3
Schmorl nodes	M51.4
Other specified intervertebral disc disorders	M51.8
Intervertebral disc disorder, unspecified	M51.9
Spinal instabilities	M53.2
Sacrococcygeal disorders, not elsewhere classified	M53.3
Other specified dorsopathies	M53.8
Dorsopathy, unspecified	M53.9
